# Structural Insights into the Substrate Egress Pathways Explains Specificity and Inhibition of Human Glucose Transporters (GLUT1 and GLUT9)

**DOI:** 10.1021/acsptsci.5c00209

**Published:** 2025-05-15

**Authors:** Manming Xu, Jiwen Jiang, Lin Gao, Saleh O. Alyemni, Shozeb Haider

**Affiliations:** † UCL School of Pharmacy, University College London, London WC1N 1AX, U.K.; ‡ University of Tabuk (PFSCBR), Tabuk 71491, Saudi Arabia; § UCL Center for Advanced Research Computing, University College London, London WC1H 9RL, U.K.

**Keywords:** GLUT1, GLUT9, pathways, glucose, urate, apigenin

## Abstract

Glucose transporters (GLUTs) play critical roles in cellular energy homeostasis and substrate-specific transport. Dysfunctional mutations can cause GLUT1 deficiency syndrome, and excessive expression of GLUT1 is linked to cancer progression, while abnormal regulation of urate transport by GLUT9 is associated with hyperuricemia and gout. In this study, machine-learning-driven molecular dynamics simulations have been employed to investigate the mechanistic insights into the substrate egress pathways of GLUT1 and GLUT9, including the inhibition mechanism of GLUT9 by apigenin. Our findings reveal that intracellular helices play a crucial role in facilitating the transition from inward-closed to -open conformations in both transporters. Additionally, aromatic residues, F_291_ and W_388_ in GLUT1 and W_336_ and F_435_ in GLUT9, are identified as key mediators of conformational changes. Analysis of substrate exit pathways provides mechanistic insights into transport profiles and aligns with clinically observed mutations. Furthermore, the inhibitory effect of apigenin on GLUT9 is shown to arise from steric hindrance due to increased substrate size rather than stable interactions. These findings enhance our understanding of GLUT transporter dynamics and highlight the potential of targeting substrate pathways for therapeutic intervention.

## Graphical abstract

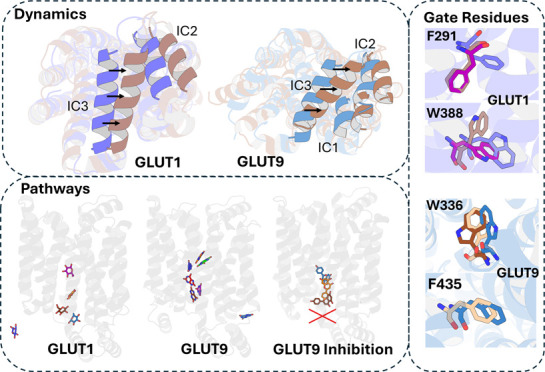

Glucose transporters (GLUTs) are a family of membrane proteins that facilitate the movement of glucose and other substrates across cell membranes, playing essential roles in cellular metabolism and homeostasis.^1^ Among the 14 members of the GLUT family encoded by the *SLC2A*, GLUT1 and GLUT9 have attracted significant attention due to their distinct substrate specificities and physiological functions.^2^ GLUT1, widely expressed across various tissues, is primarily responsible for basal glucose uptake, which is crucial for energy supply in metabolically demanding organs such as the brain and erythrocytes.^2^ In contrast, GLUT9 exhibits unique functional characteristics extending beyond glucose transport to play a critical role in urate handling. Dysregulation of urate transport by GLUT9 is implicated in hyperuricemia and gout.^3,4^ This has led to a focus on research and development of drugs that target GLUT9 and competitively inhibit its binding to urate, such as apigenin.^5^

Both GLUT1 and GLUT9 consist of 12 transmembrane α-helices (Figure 1A,B). Interestingly, although GLUT1 and GLUT9 share a significant similarity in structural conformations, they share only a 39.37% sequence similarity (Figure 1C). The difference is that GLUT9 is enriched in polar residues and has 5 intracellular helices, whereas GLUT1 is enriched in hydrophobic residues and has only 4 intracellular helices (ICs).^5–7^ Moreover, compared with GLUT1, which exclusively transports glucose, GLUT9 can transport both glucose and urate. Kinetic studies using Xenopus oocytes have shown that GLUT9 mediates urate uptake (*V*_max_ ≈ 304 pmol/oocyte/20 min), whereas glucose transport under similar conditions is significantly lower, corresponding to a 45–60-fold higher transport rate for urate.^8^ However, the structural mechanisms for this specificity remain poorly understood. The recent publication of the cryo-EM structures of human GLUT9 in complex with urate and the flavonoid inhibitor apigenin^5^ has elucidated the structural basis and the critical residues involved in urate transportation and apigenin inhibition, highlighting key aspects of ligand binding. However, Shen et al.^5^ failed to provide information on the dynamic processes essential for the transporter function, thus limiting our understanding of the specific mechanisms of ligand selection.

**Figure 1:**
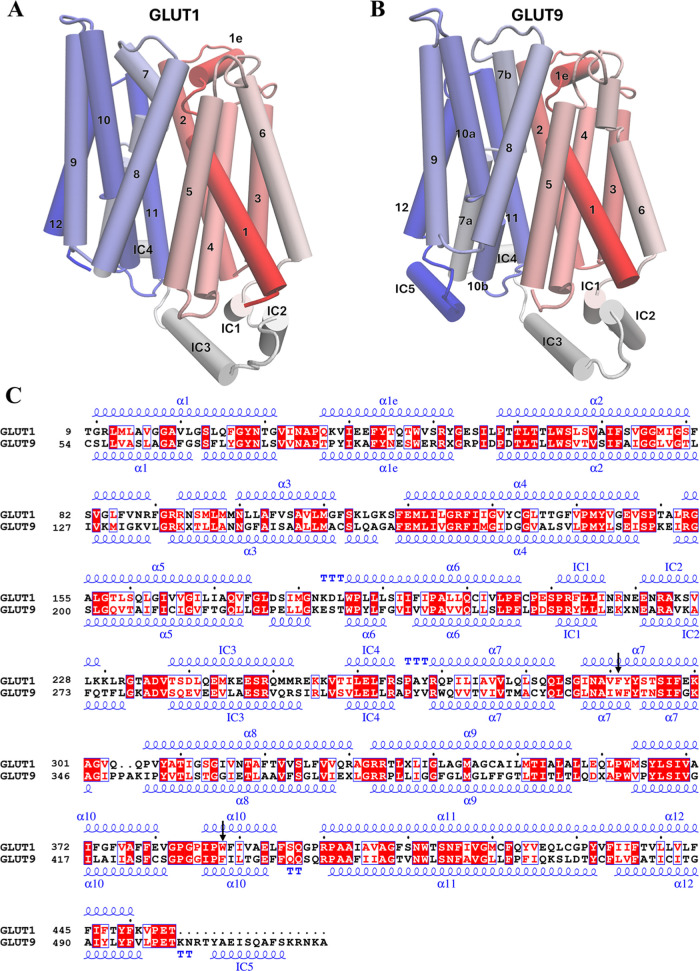
(A) Structure nomenclature of GLUT1, containing 12 transmembrane helices and 4 intracellular helices. (B) Structure nomenclature of GLUT9 consisted of 12 transmembrane helices and 5 intracellular helices. (C) Sequence alignment of GLUT1 and GLUT9, with a sequence similarity of 39.37%. Residue pairs pointed by the black arrows are the structurally equivalent aromatic residues in GLUT1 and GLUT9.

Molecular dynamics (MD) simulations offer a powerful means to study protein dynamics and protein–ligand interactions, revealing critical conformational changes and key pathways in substrate translocation.^9,10^ The Markov state model (MSM) is a method widely used in combination with MD simulations to study the long-time scale conformational dynamics of biomolecules.^11^ In some systems, traditional MSMs require long lag times to achieve Markovianity between states. This limits temporal resolution,^12^ especially for systems like GLUTs where slow transitions and long-lived intermediate states exhibit non-Markovian behavior. To overcome this challenge, we applied a non-Markovian modeling approach using the recently introduced integrative generalized master equation (IGME) to investigate GLUT1 and GLUT9 dynamics and apigenin inhibition of GLUT9. IGME incorporates memory effects to accurately model non-Markovian dynamics without the need for long lag times.^13^ In conjunction with the IGME, we employed PathDetect-SOM, a neural network-based tool that employs self-organizing maps (SOMs) to cluster conformations from MD simulations and map ligand-binding pathways in a 2D space.^14^ PathDetect-SOMs enable unsupervised identification of ligand pathways directly from unbiased molecular dynamics simulations, without requiring predefined reaction coordinates or ligand poses, and thereby allow us to trace and compare the egress pathways of urate and glucose in both GLUT1 and GLUT9.

By integrating PathDetect-SOM with IGME, we achieved a detailed understanding of dynamics and substrate specificity in GLUT9 and GLUT1 across various ligand-bound and inhibitor-bound states. These insights identify molecular determinants driving GLUT9 function, advancing our knowledge of substrate recognition mechanisms within the GLUT family, and supporting the development of targeted therapies for metabolic disorders linked to urate and glucose transport dysregulation.

## Results

### Non-Markov State Models (nMSMs)

Three converged non-Markov state models (nMSMs) were constructed for GLUT1 apo, GLUT9 apo, and GLUT9 API (apigenin binding) separately. The feature applied to each system is identical, using the χ_1_ angle of selected binding essential residues and the distance between helices (details in Supporting Information). Ten metastable states were extracted for GLUT1 apo at a lag time of 5 ns (Figure S1A,C). State 1 is the highest energy state in GLUT1, and state 10 is the lowest energy state, accounting for 28.3% of the overall trajectory (Figure S1D). The flux analysis shows that the most frequent pathway from state 1 to state 10 is 1–3–7–8–10 (Figure S1C). The model shows a good alignment with the simulation data in the Chapman–Kolmogorov (CK) test (Figure S1E), indicating that the nMSM accurately captures the long-time scale dynamics of the system. The mean first passage time (MFPT) was calculated based on the prediction of IGME. MFPT is not fully consistent with the flux network, as we are dealing with the non-Markovian process (Figure S1F).

Similarly, 7 metastable states were separated from the GLUT9 apo system at a lag time of 2 ns (Figure S2A,C), with state 1 being the highest energy state and state 7 being the lowest (Figure S2D). The flux analysis indicates that the most frequent pathway from state 1 to state 7 passes through state 4, which is a high-energy intermediate (Figure S2C). The flux result aligns with the estimated MFPT among states (Figure S2F). For the apigenin-binding system of GLUT9, 6 metastable states were observed, with the most frequent pathway detected as 1–4–6 (Figure S3C) at a lag time of 2 ns (Figure S3A). The MFPT results are consistent with the flux analysis (Figure S3F). Both models have passed the CK test, confirming their reliability (Figures S2E and S3E). A more detailed description of the parameters used to build the nMSM is presented in the Methods section.

### Intracellular Helices, F291, and W388 Are Vital for GLUT1 Dynamics

Metastable states in GLUT1 Apo can be clustered as outward-open, intermediate, and inward-open conformations based on the solvent accessible surface area (SASA) value of the extracellular and intracellular sides. The outward-open conformation would have a higher SASA value on the extracellular side and a lower SASA value on the intracellular side, and vice versa. The SASA result suggests that the most frequent pathway in this system describes the protein dynamics change from outward-open (state 1) to a relatively stable intermediate (state 8) and eventually reaches the lowest energy inward open (state 10). State 1 has the largest extracellular side SASA, suggesting that it is more outward-open (Figure 2A). Conversely, state 10 has the largest intracellular side SASA, implying that it is more inward-open (Figure 2B).

**Figure 2:**
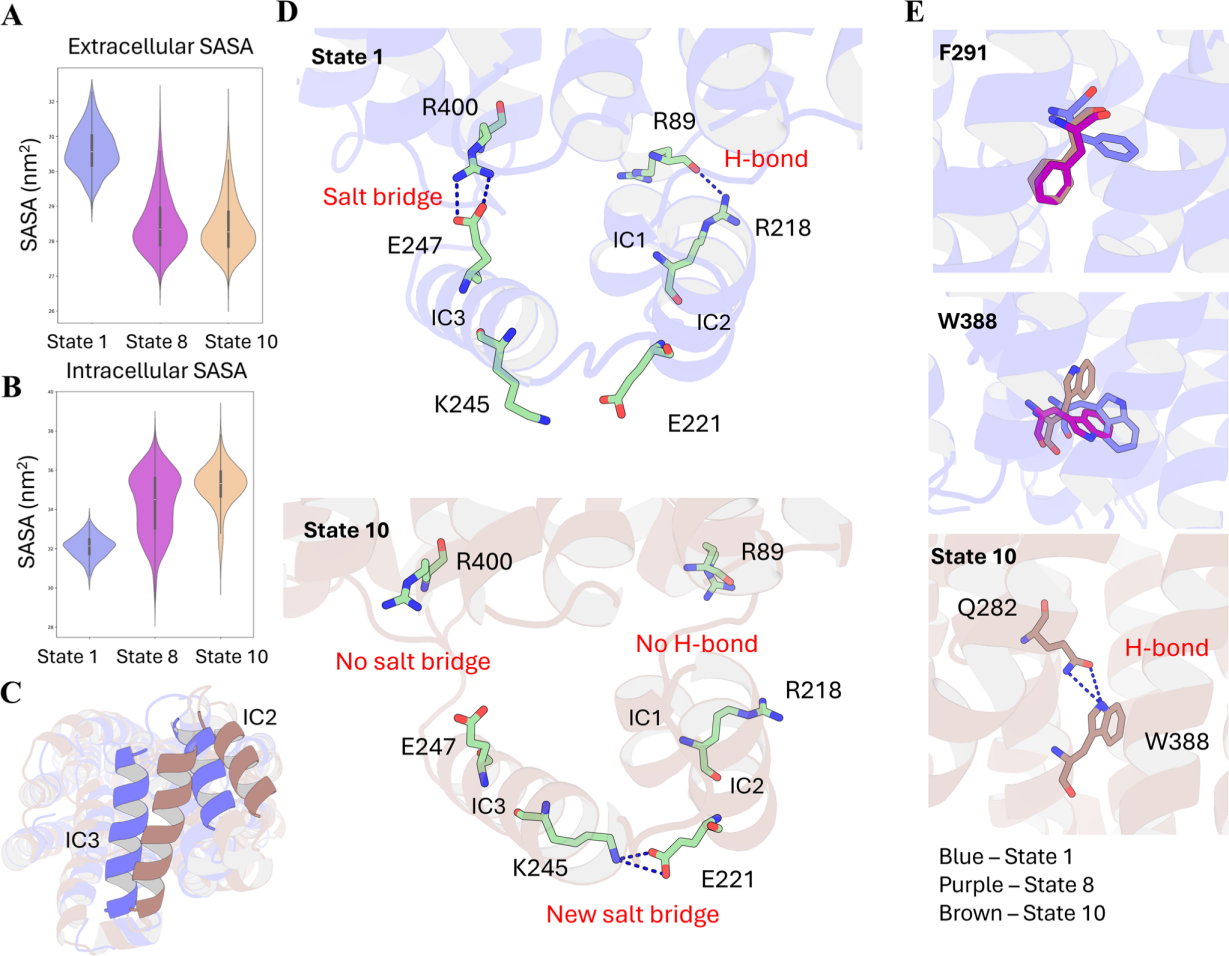
GLUT1 nMSM Analysis. (A) SASA value of the extracellular side. (B) SASA value of the intracellular side. (C) Structure alignment of state 1 and state 10 (intracellular view), where IC3 and IC2 undergo significant movements. (D) In the inward close state 1, R_400_ and E_247_ can form a stable salt bridge, and R_89_ and R_218_ are hydrogen bonds to each other. No interaction can be observed between E_221_ and K_245_. In the inward open state 10, previously witnessed interactions are completely lost, while a steady new salt bridge is formed between E_221_ and K_245_. (E) Conformation change of F_291_ and W_388_ among the three states and the H-bond interaction formed between Q_282_ and W_388_ in state 10. Blue—state 1, purple—state 8, and brown—state 10.

Superimposition of states 1 and 10 illustrates significant movements among the intracellular helices (ICs), especially IC2 and IC3 (Figure 2C). In state 1, IC3 blocks the tunnel, while in state 10, IC3 flips remarkably and leads to the opening of the intracellular side (Figure 2C). Thus, understanding the movement of these ICs becomes essential, as they correspond to the open and close dynamics. Several reasons have been identified that trigger the motion of IC3. First, the loss of interaction between IC3 and TM11 frees IC3 and enables its further movements. R_400_ (TM11) and E_247_ (IC3) form a strong salt bridge and hydrogen bond interactions in state 1 (Figure 2D). Distance calculation indicates that the side chain between these two residues is within 4 Å in state 1 over 80% of simulation time (Figure S4A,B). However, these interactions are completely lost in state 10 (Figure 2D), where the residence time of these interactions decreases to approximately 5% (Figure S4A,B). Moreover, the flip of IC1 makes more space and contributes to the movement of IC3. IC1 maintains its position in state 1 through a hydrogen bond interaction between the R_89_ backbone (TM2) and the R_218_ side chain (IC1) (Figure S4D) with over 60% residence time (Figure S4C). In state 10, this hydrogen bond is much less likely to occur (Figures 2D and S4C). Lastly, the strong salt bridge and hydrogen bond between K_245_ (IC3) and E_221_ (IC2) drag IC3 to move toward the N terminal of helix 1, leading to the significant movement of this secondary structure (Figure 2D). The carboxylate group of E_221_ shows very close contact with side chain ε-nitrogen of K_245_ in state 8 and state 10, based on the distance calculation, indicating strong interactions (Figure S4D,E). These factors collectively drive IC3 to move closer to the N terminal of helix 1, leading to the opening of the intracellular side (Figure 2D).

Furthermore, two residues with aromatic ring structures are identified as essential for the transporter.^15,16^ F_291_ is situated at the extracellular gate. The χ_1_ angle distribution of F_291_ indicates that the two high-energy states (state 1 and state 2) prefer a trans(180°) orientation, which is significantly different from other states (Figure S5A). All the other 8 states, which are more energetically favorable, have a gauche­(+)(−60°) conformation (Figure S5A). This results in a different orientation of F_291_ (Figure 2E). F_291_ has been identified in previous studies as a tracker of glucose,^15^ as mutations on this site significantly alter the glucose transport ability of GLUT1.^17^ The horizontally placed side chain in state 1 enables the tracking of glucose via the aromatic ring, indicating that the extracellular side is actually open. However, in states 8 and 10, the F_291_ side chain is placed alongside the TMs (resting state), losing the ability to attract glucose from the extracellular environment. On the intracellular side, W_388_ has been found to be essential for the conformation change from state 8 to state 10, whose χ_1_ angle corresponds to the separation in the free energy plot (Figure S5A). W_388_ in previous studies has been shown to be essential for the conformational change of GLUT1 from outward-open to inward-open.^16^ In state 1 and state 8, the χ_1_ angle of W_388_ is around trans(180°), while in state 10, it changes to gauche­(+)(−60°), where the side chain of the residue rotates, resulting in the opening of the tunnel (Figure 2E). Q_282_ was found to significantly contribute to this orientation of W_388_ by forming strong hydrogen bonds that stabilize the W_388_ side chain (Figure 2E). A remarkable residence time of this interaction in state 10 is confirmed by the distance calculation (Figure S5B). Moreover, W_388_, together with P_141_, M_142_, and I_404_, forms a hydrophobic gate that blocks the tunnel in state 1. The rotation of the W_388_ side chain sterically pushes the residues apart in state 10, thereby opening the tunnel’s inward side (Figure S5C).

### GLUT9 Shows Similar Dynamic Behaviors to GLUT1

The partial SASA value is used to define the outward-open and inward-open conformations in GLUT9. The SASA value result indicates the nMSM dominant pathway (1–4–7) describes the transition from an inward-close conformation (state 1) to an inward-open conformation (state 7), as state 7 has a higher value of intracellular SASA (Figure 3B). However, unlike GLUT1, which witnesses an inverse in the SASA value of the two sides, the opening and closure of the extracellular side in GLUT9 do not seem to affect its SASA value (Figure 3A).

**Figure 3:**
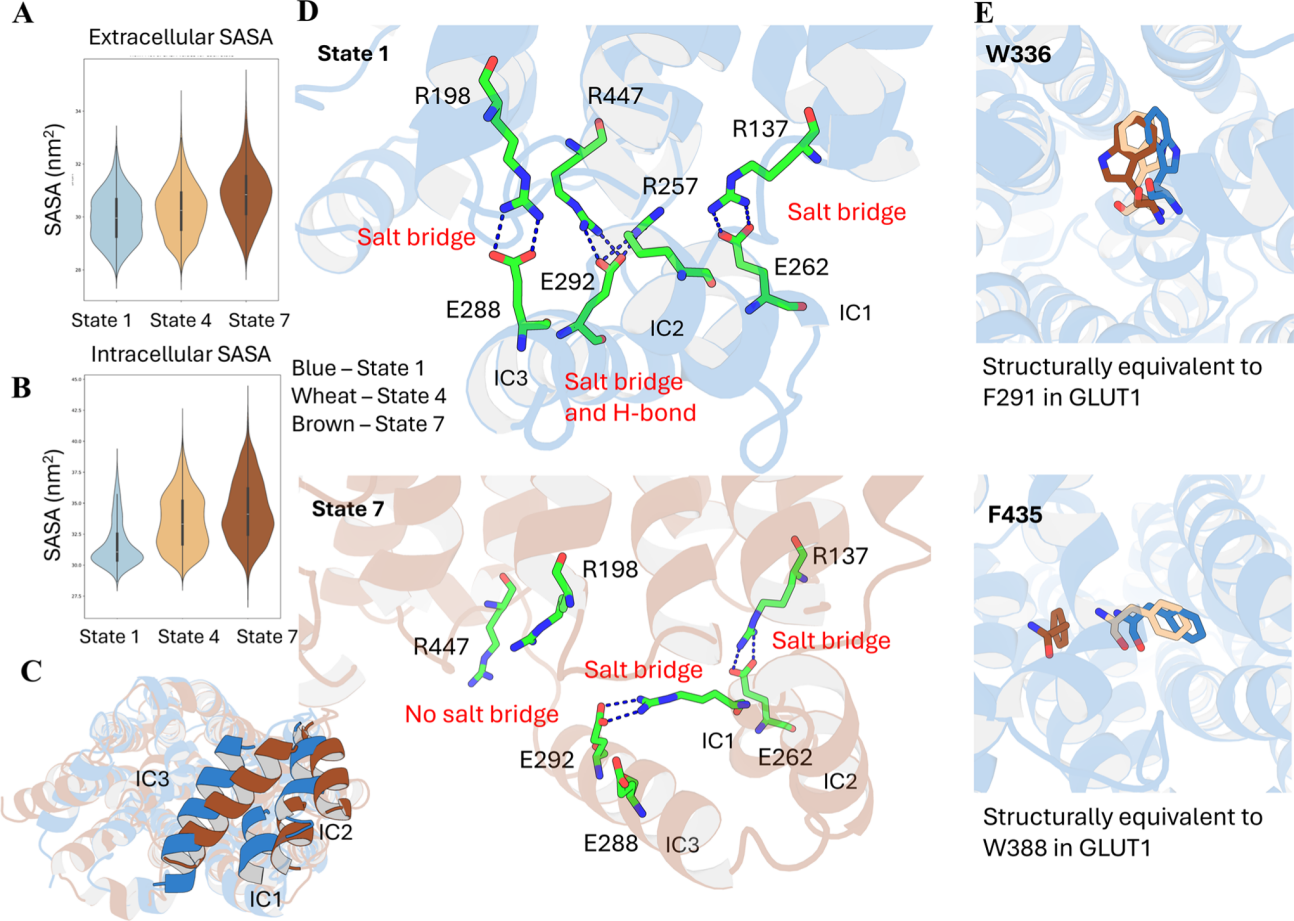
GLUT9 nMSM Analysis. (A) SASA value of the extracellular side. (B) SASA value of the intracellular side. (C) Structure alignment of state 1 and state 7 (bottom view), where ICs undergo significant movements. (D) In the inward close state 1, four salt bridges could be observed: R_137_–E_262_, R_198_–E_288_, R_257_–E_292_, and E_292_–R_447_. In the inward open state 7, salt bridges R_198_–E_288_ and E_292_–R_447_ decrease occurrence, while the other two salt bridges preserve. (E) Conformation change of W_336_ and F_435_ in each state. Blue—state 1, wheat—state 4, and brown—state 7.

Similar to GLUT1, GLUT9 undergoes tremendous movement in its ICs, especially IC3, where they move closer to the N terminal of helix 1 and open the intracellular side (Figure 3C). In state 1, the ICs are held and stabilized through several salt bridges with the protein backbone (Figure 3D). E_262_ on IC1 forms a stable salt bridge with R_137_ on TM3 (80% residence time) (Figure S6A). Two salt bridges between E_288_ (IC3) and R_198_ (TM5) and E_292_ (IC3) and R_447_ (TM11) stabilize IC3 in its place in state 1, both with a residence time >60% (Figure S6B,C). One additional salt bridge between R_257_ and E_292_ links IC1 and IC3 (Figure S6D). In state 7, however, the residence time of the two salt bridges between the TMs and IC3 drops significantly, which frees IC3 and enables its further motion (Figure S6B,C). In contrast, the salt bridges that connect IC1 to TM3 and IC3 to IC1 are preserved (Figure S6A,D). Thus, with the opening of the intracellular side, IC1 would be dragged toward the N terminal of helix 1, which subsequently triggers the movement of IC3 in the same direction, ultimately leading to tunnel opening (Figure 3D).

Like in GLUT1, we identified two aromatic residues in GLUT9 acting as gate modulators. W_336_ and F_435_, which are structurally equivalent to F_291_ and W_388_ in GLUT1, undergo significant conformational changes along the pathway (Figure 3E). Unlike F_291_, which is more likely to be a binding residue, W_336_ acts as a gating residue in GLUT9. Urate is much larger than glucose and is prevented from entering the tunnel, while the side chain of W_336_ is positioned perpendicular to the transport axis (Figure S7A,B). Thus, state 4 and state 7, where the trans(180°) conformation of W_336_ is observed, are considered as outward-closed, while state 1, where the gauche(−)(60°) conformation dominates, is considered as outward-open (Figure S7C). A very similar phenomenon could also be observed at the intracellular side of GLUT9, where F_435_ modulates the opening and closure of the intracellular side. In this case, state 1 prefers a χ_1_ angle of trans(180°), where the side chain blocks the tunnel, while state 7 tends to have a χ_1_ angle of gauche(−)(60°), where the intracellular side opens (Figure S7D). State 4, however, shows a relatively equal preference for both orientations, indicating that it is an intermediate state. The significant difference in the side chain orientation as well as the position from state 1 to state 7 enlarges the tunnel on the intracellular side (Figure S7E). The observation of the difference in the conformation of these two aromatic residues aligns with the result of nMSM, indicating that the pathway successfully describes the conformation change of GLUT9 as well as emphasizes the significant contribution of these two residues to GLUT9 apo dynamics.

### GLUT1 Glucose Pathway Explains Its Preference

For the exit of glucose, seven clusters were identified by PathDetect-SOM.^14^ According to the distance heatmap, glucose shows the least contact with the selected residues in cluster A, while it shows the most contact in cluster E (Figure 4A). Thus, cluster E is considered the initial binding state, and cluster A represents the completely unbound conformations. Two possible pathways, all starting from cluster E to cluster A, are illustrated in the neuron transition network: E (purple)–C (red)–B (green)–A (blue) and E (purple)–D (orange)–F (brown)–G (sky blue)–A (blue) (Figure 4B). At the starting point in cluster E, glucose forms close contact with residues P_26_, Q_161_, Q_283_, N_288_, Y_292_, F_291_, N_411_, and N_415_, which is consistent with the conformation observed in the crystal structure (PDB id: 4PYP^7^) (Figure S8A).

**Figure 4:**
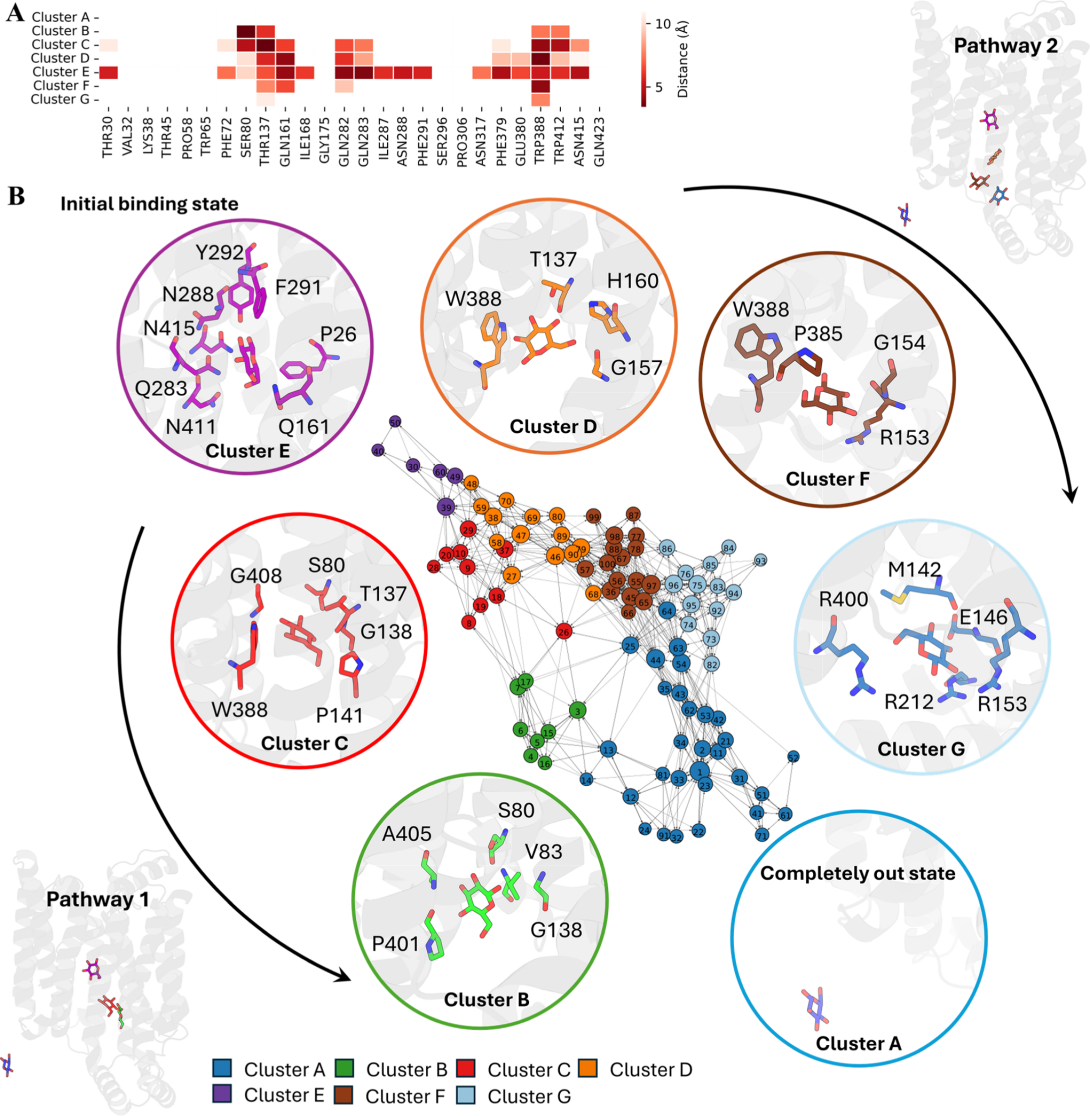
GLUT1 glucose pathway results. (A) Distance heatmap between glucose and selected input residues during SOM training. (B) Transition network with potential pathways indicated by black arrows. Detailed ligand–protein contacts in each cluster are placed close to the neuron with residues within 4 Å of the substrate highlighted; each pathway follows the arrow from cluster E to cluster A.

In the first pathway, glucose moves out from the crevice between TM2 and TM11 (Figure S8B). In cluster C, S_80_, T_137_, G_138_, and W_388_ form contacts with glucose (Figure 4B). After passing S_80_, glucose enters a site surrounded by residues with short side chains: V_83_, G_138_, and A_405_ (Figure 4B). Thus, the protein is not able to hold glucose, and glucose exits in the next stage. This exit position does not seem to be influenced by the movements of the ICs but depends on the distance between TM2 and TM11 on the intracellular side. In the inward-closed structure defined by nMSM previously (state 1), the crevice between TM2 and TM11 is quite narrow, thus eliminating the possibility of the escape of glucose (Figure S8C). An alternative exit position is also observed in pathway 2, where glucose moves out of the tunnel opened by the movement of IC3 (Figure S8D). Glucose first shows very close contacts with T_137_, G_157_, H_160_, and W_388_. After cluster D, glucose slides vertically into a hydrophobic environment formed by R_153_, G_154_, P_385_, and W_388_. It then follows a relatively polar path surrounded by M_142_, G_145_, E_146_, R_153_, R_212_, and R_400_. Unlike the first pathway, this route is strongly modulated by the movement of ICs, especially IC3. Superimposition of the structures indicates that glucose is not able to exit the protein through this pathway in the inward-closed conformation defined by the nMSM model (state 1), as the tunnel is blocked by IC3 (Figure S8C). W_388_, an important residue we mentioned in GLUT1 apo dynamics, contributes to both glucose pathways with its side chain flipped up, opening the intracellular gate as we proposed (Figure 4B).

Compared with glucose, urate is much larger and contains one additional ring structure. In nature, the GLUT1 transporter does not transport urate.^5^ Ten clusters were identified when analyzing the urate exit pathway of GLUT1 (Figure S9). From the distance heatmap, cluster H is identified as the unbound state (Figure S9A). However, to identify the bound state of urate, where in clusters C, D, and E all show close contacts between the substrate and the protein, was challenging. From the neuron transition network, cluster D (orange), which is located at one edge of the network, is assumed to be the initial binding stage (Figure S9B). Unlike glucose, the transit of urate does not follow a clear route to exit, as it tumbles inside the tunnel (Figure S9B). Cluster B and cluster F are indistinguishable and display an unstructured transition network (Figure S9B). Detailed contact conditions in these clusters suggest that the urate is held in a very similar place in these two clusters, where W_388_ can form a strong π–π interaction with the urate (Figure S9B). This interaction is not observed in the glucose system as glucose lacks an aromatic ring and the structural features necessary for aromatic stacking. Thus, the rotation of the side chain of W_388_ only opens the tunnel in the glucose system and does not strongly influence its exit. Compared to the glucose system (glucose cluster A), the number of frames in which urate is in the completely unbound state (urate cluster H) is more than three times lower, suggesting a much slower exit. The unstructured pathway of urate in GLUT1 explains why GLUT1 cannot transport urate.

### GLUT9 Urate Pathway Explains Its Preference

Unlike GLUT1, which exclusively transports glucose, GLUT9 can transport both glucose and urate but has a 50 times preference for urate.^18^ Thus, understanding why this preference happens is quite essential to understanding the transport mechanism of GLUT9. Pathway analysis provided a clear exit pathway for the urate. Detailed contact information illustrates constant interactions between urate and GLUT9 (Figure 5). PathDetect-SOM deduced two unique binding conformations for urate in GLUT9, cluster B and cluster D. In cluster B, the side chain of R_171_ interacts with urate via hydrogen binding. Residues with aromatic ring structures appear in the neighborhood but do not participate in any significant interactions. Another binding conformation, which is more favorable, is cluster D, which shows more stable interactions. W_336_, an essential residue acting as a gate on the extracellular side of GLUT9, forms a π–π interaction with the indole ring of the urate. Moreover, N_79_ and E_364_ form hydrogen bonds with urate. GLUT9 favors cluster D, as the frames belonging to cluster D are three times more than that of cluster B. Urate then follows a single exit pathway: A (blue)–C (red)–E (purple)–F (brown)–G (sky blue) (Figure 5B). After passing W_336_, the urate forms hydrogen bond interactions with the side chain of Y_327_, Q_328_, N_333_, W_336_, and E_364_ (cluster A). In the next stage (cluster C), all of the hydrogen bond interactions are lost except for that with Y_327_. F_435_ then takes part in and forms a π–π interaction to urate. F_435_ is structurally equivalent to W_388_ in GLUT1. However, the interaction it forms might be weaker compared to tryptophan, as the tryptophan indole ring provides a larger, more complex interaction surface that can engage more extensively in stacking or hydrophobic contacts. Such a relatively weaker response might allow GLUT9 to balance the role of directing urate and transporting it into the cell. After passing F_435_, the urate then loses all of the interactions with the protein in cluster F, thus going out of the transporter in the next stage (Figure 5B).

**Figure 5:**
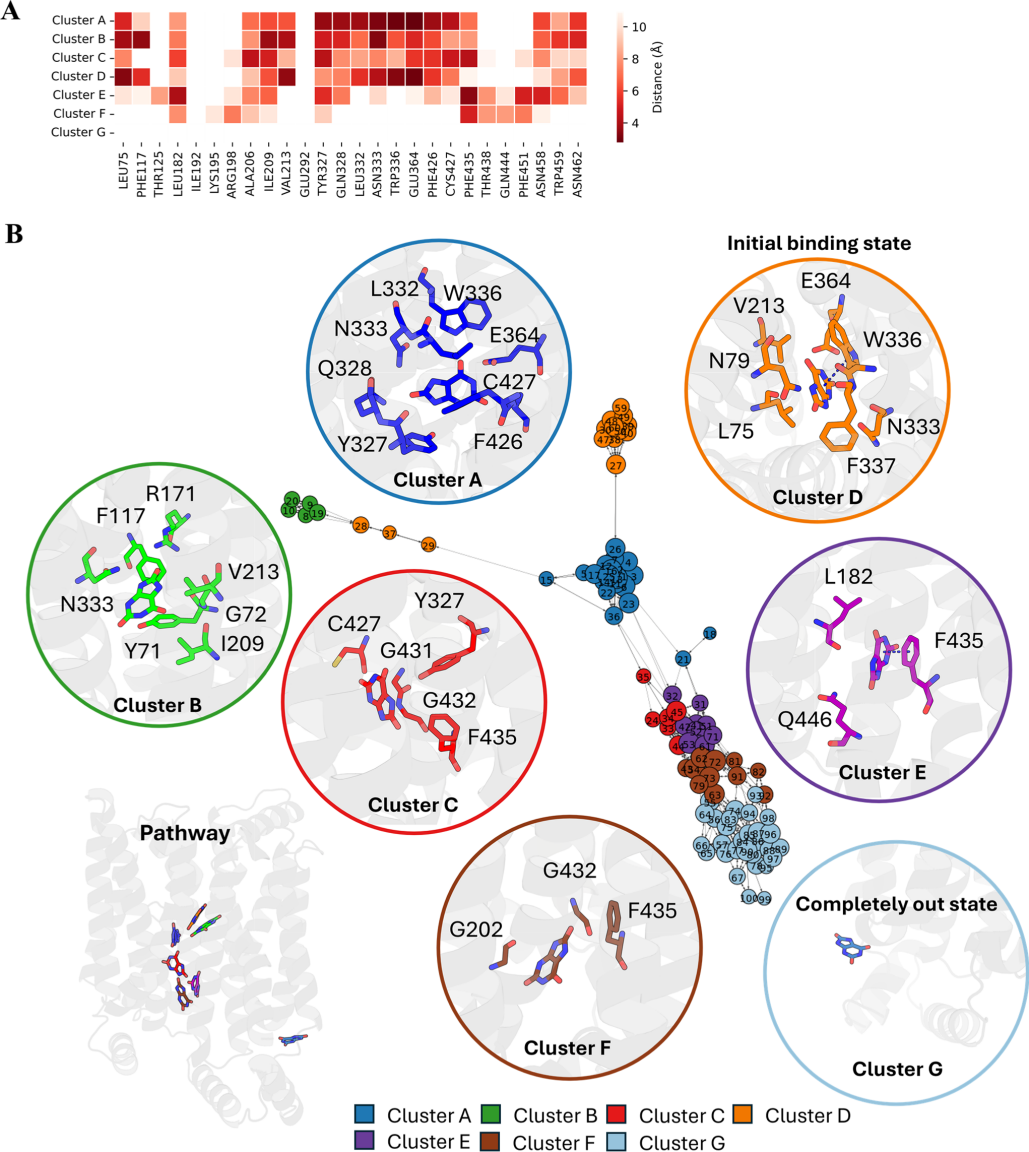
GLUT9 urate pathway results. (A) Distance heatmap between glucose and selected input residues during SOM training. (B) Transition network. Detailed ligand–protein contacts in each cluster are placed close to the neuron with residues within 4 Å of the substrate highlighted; the pathway starts from cluster D and ends at cluster G.

Compared to urate, glucose is smaller and more hydrophilic, surrounded by oxygens that easily form interactions with the polar side chains alongside the tunnel. As mentioned previously, GLUT9 is more enriched in hydrophilic residues compared with GLUT1. The increased potential interactions between GLUT9 and glucose due to increased substrate hydrophilicity might be one of the reasons for the preference of GLUT9 to urate. Nine clusters were defined by PathDetect-SOM, and the distance heatmap indicates a very close contact between glucose and GLUT9 in clusters A, B, C, and D. Cluster I is considered the completely unbound state for glucose according to the heatmap (Figure S10A). Acting as another edge of the transition network, cluster B is defined as the starting bound conformation of glucose. Unlike urate, which exhibits a clear exit pathway, the transition network of glucose is much more complex, indicating multiple interaction sites inside the tunnel. In cluster B, polar side chains of Y_71_, Y_327_, Q_328_, and N_333_ are able to form interactions with glucose, which is quite similar to what happens in urate cluster A. However, compared to the urate case, the interactions between glucose with W_336_ or E_364_ are completely lost in the initial state. After the starting state is left, rather than following a direct pathway, glucose tumbles inside the tunnel. Its position in clusters A, C, and D is quite similar (Figure S10C). Moreover, in cluster B to cluster A or cluster B to cluster C, glucose moved slightly closer to the extracellular side. Q_328_, N_333_, W_336_, and E_364_, which are essential for the exit of urate, also play roles in the glucose pathway. These residues form interactions with glucose throughout the first several clusters (A, B, C, and D). However, as glucose only contains oxygen, some interactions formed by these residues are much weaker compared to those with urate. This might be one of the plausible reasons that cause the differences in substrate preference. Cluster E becomes a critical intermediate state that connects the four initial clusters (A, B, C, and D) and the four subsequent clusters (F, G, H, and I) (Figure S10B). Detailed conformation analysis implies a relatively hydrophobic environment within cluster E, with glucose surrounded by hydrophobic residues: L_182_, P_186_, P_434_, F_435_, and I_436_. After passing cluster E, glucose continuously forms close contacts with the protein until it leaves cluster I. However, urate is much less likely to be sequestered in complex sites, such as glucose, after leaving its initial binding state, which would explain the structured exit pathway of urate. Moreover, π–π stacking plays an important role in guiding the urate. F_435_, which we proposed as a gate residue having a similar function as W_388_ in GLUT1, forms π–π with urate, providing stability to prevent its rotation inside the tunnel. In contrast, the π–π stacking would all be lost in the case of glucose, resulting in the tumbling and rotation of the substrate around F_435_, as shown in clusters E, F, and H.

### GLUT9 API Dynamics Explains the Inhibition

Apigenin binding to GLUT9 was simulated to study the dominant binding position of this inhibitor inside GLUT9. The most dominant pathways in this system is 1–4–6. Superimposition between state 1 and the cryo-EM structure shows a very similar apigenin binding position (Figure 6A), indicating the cryo-EM binding state is our starting point in this deduced pathway. The most significant differentiation on the free energy map from left to right is caused by the χ_1_ angle of W_336_, an important residue that also undergoes significant conformation changes in GLUT9 apo dynamics (Figure 6B). The horizontally positioned side chain of W_336_ in state 1, state 2, and state 4 blocks the tunnel on the extracellular side, indicating an extracellular closed conformation (Figure S11A). This χ_1_ angle, however, prefers a completely different orientation in the other three states, where the side chain flips down toward the intracellular side and opens the extracellular side (Figure S11A). Thus, these states are more likely to be an outwardly open conformation. The apo dynamics of GLUT9 indicate that the transporter prefers an inward-open conformation, as both the cryo-EM structure and the extracted lowest energy conformation from nMSM are all inward-open. However, with the movement of apigenin, the transporter transforms from an energetically preferred inward-open conformation to a high-energy outward-open conformation.

**Figure 6:**
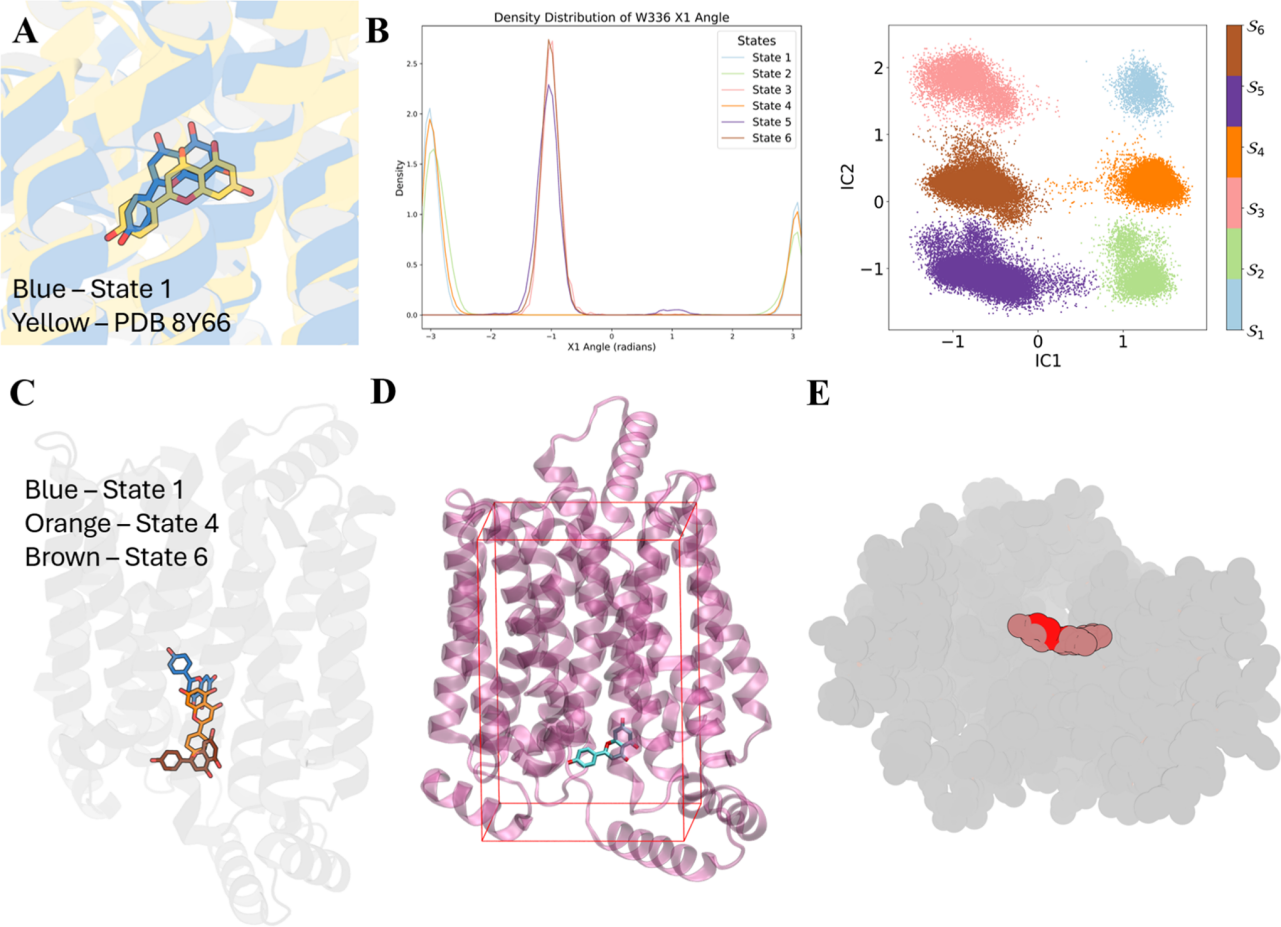
(A) Superimposition of extracted state 1 and original PDB 8Y66.^5^ (B) The χ_1_ angle of W_336_ and the corresponding state distribution map. (C) Ligand pathway. (D) Defined flat-bottomed restraints and extracted structure from state 6. (E) Sphere representation of GLUT9 and API.

Moreover, unlike urate and glucose exiting the GLUT9 tunnel during simulations, apigenin was retained within the GLUT9 tunnel without touching the restraints (Figure 6D). We initially hypothesized that GLUT9 might form more and stronger interactions with apigenin potentially via multiple hydrogen bonds. To explore this, representative structures were extracted from the energy minimum of each metastable state, and potential hydrogen bond interactions were identified. However, within these proposed interactions, no pair of atoms maintained a proximity of less than 4 Å with occupancy above 50%, suggesting that no consistent, close–contact interactions primarily account for the retention of apigenin even in the final stable position state 6 (Figure S11B).

Therefore, we attribute this retention to the macroscopic structural characteristics of the GLUT9-apigenin complex. We propose that the relatively large and rigid structure of apigenin perturbs the conformational equilibrium of GLUT9, shifting it from an inward-open state to an outward-open state. In this conformation, the intracellular gate is closed, and apigenin remains trapped near the intracellular side. The sphere representation of state 6 (Figure 6E) illustrates apigenin’s buried position and the absence of a viable exit path, consistent with a steric retention mechanism rather than stable binding.

## Discussion

### GLUT1 and GLUT9 Share Similar Dynamic Behaviors

GLUT1 and GLUT9 are structurally and dynamically similar to each other. In both proteins, the ICs, especially IC3, determine the opening of the intracellular side through the modulation of a series of salt bridges formed either between ICs or between ICs and TMs. Both proteins contain two dynamically critical aromatic residues, F_291_ and W_388_ in GLUT1 and W_336_ and F_435_ in GLUT9, which are structurally equivalent. F_291_ in GLUT1 tracks glucose and thus needs to orient in a trans(180°) conformation to expose its side chain to the solvent. On the other hand, W_388_ in GLUT9 and W_336_ and F_435_ in GLUT9 act as gate modulators that strongly correspond with the open and closed conformations on each side.

### Pathway Detection Explains Substrate Preference

Glucose and urate pathways in each protein have been analyzed, revealing distinct substrate preferences. GLUT1 is incapable of transporting urate, whereas GLUT9 demonstrates a 50-fold preference for urate over glucose.^18^ The desired substrate in each case presents a structured pathway. Despite the dynamic similarities between the two proteins, differences in the chemical profile of the transport tunnel and the intracellular exit site significantly influence substrate selectivity. In GLUT1, the potential strong π–π interaction formed between W_388_ and urate disrupts the urate pathway. In contrast, the substitution of W_388_ with F_435_ in GLUT9 results in a smaller and less chemically complex side chain, which may weaken its interaction with urate. This alteration facilitates the smoother release of urate while providing additional stabilization that minimizes substrate misorientation.

Mutations affecting the transport ability of both transporters were mapped along the identified pathways. In GLUT1, cysteine-scanning mutagenesis showed that E_146_C reduced transport activity by 97%, indicating a critical functional role.^19^ Clinically, missense mutations at T_137_, M_142_, E_146_, and R_153_ have been reported in GLUT1 deficiency syndrome.^21^ The human cerebrospinal fluid (CSF) blood glucose ratio is around 0.6.^20^ Substitutions T_137_A, M_142_K, and R_153_H moderately reduced CSF/blood glucose ratios (0.56, 0.4, and 0.44, respectively), while E_146_K caused a severe drop to 0.25.^21^ Strikingly, all four residues lie within cluster G and are the final state before intracellular glucose release. In GLUT9, three clinically reported mutations, namely, L_75_R, R_171_C, and N_333_S, were found along the identified urate transport pathway. While R_171_C shows minimal impact on urate uptake, L_75_R and N_333_S significantly reduce transport efficiency.^22^ R_171_ and N_333_, both polar residues, appear in the early clusters of the uptake pathway, suggesting a role in stabilizing the urate-binding conformation during midtransport. L_75_ is hydrophobic; its substitution to arginine introduces additional hydrogen bonding interactions (Figure 5, cluster D), which may disrupt the correct substrate orientation and impair uptake efficiency. These spatial convergences across GLUT1 and GLUT9 highlight conserved mechanistic roles of clinically mutated residues in facilitating substrate translocation, consistent with their observed functional and pathological impacts.

### API Acts as a Plug to GLUT9

Apigenin is a natural product that has been found as a GLUT9 inhibitor.^23^ Previous studies and the published cryo-EM structure indicate several hydrogen bond interactions that anchor apigenin inside the transporter.^5^ However, based on our dynamic analysis, no consistent interactions could be formed between GLUT9 and apigenin. The sphere representation of the lowest energy structure suggests that apigenin inhibits the transporter purely due to its size and acts as a plug that blocks the GLUT9 tunnel.

## Conclusion

In conclusion, our study compares the apo dynamics of GLUT1 and GLUT9, emphasizing their dynamical similarities. We analyzed the exit pathways for glucose and urate in both transporters, explaining the preference of urate to glucose in GLUT9, as well as the inhibition mechanism of GLUT9 inhibitor apigenin. These findings enhance our understanding of the substrate selectivity and regulatory mechanisms of these transporters, especially those for GLUT9. A key limitation of this study is that our simulations were initiated from the inward-opening cryo-EM structure, which precluded observation of the extracellular entry process. Additionally, while our analysis highlights a structural difference at the intracellular gate (W_388_ in GLUT1 vs F_435_ in GLUT9) that correlates with distinct egress behavior, we cannot definitively attribute the absence of urate transport in GLUT1 to this residue alone. Other factors, such as substrate entry dynamics, global conformational changes, or differences in binding affinity, may also contribute and were not captured in our simulations. Future studies integrating outward-open or occluded conformations will be necessary to capture the complete transport cycle, including substrate recognition and entry, to better inform therapeutic targeting strategies.

## Methods

### Building the GLUT Systems

The cryo-EM structures of the membrane proteins GLUT1 (PDB: 4PYP^7^) and GLUT9 (PDB: 8Y65 ^5^ and 8Y66 ^5^) were obtained from the PDB. The HTMD Python package^24^ was used to build seven systems (details in Table 1) as follows: GLUT1-Apo, GLUT1-Glucose (with glucose in the binding site), GLUT1-Urate (with urate in the binding site), GLUT9-Apo, GLUT9-Glucose (with glucose in the binding site), GLUT9-Urate (with urate in the binding site), and finally GLUT9-API (with apigenin in the binding site). The apo systems were generated directly from the experimental structures by removal of the ligands. For the GLUT1-Glucose system, we extracted the glucose core from the cocrystallized ligand nonyl β-d-glucopyranoside by removing the bulky spiro tail from PDB entry 4PYP.^7^ The GLUT9-Glucose system was constructed by superimposing GLUT9 onto the GLUT1-Glucose complex and transferring glucose coordinates. A similar approach was applied to build the urate-bound systems from PDB entry 8Y65.^5^ The apigenin-bound structure of GLUT9 was obtained directly from PDB entry 8Y66.^5^ The protonation states of the titratable residues were predicted based on pH 7 using PROPKA3 and PDB 2PQR as implemented in the HTMD function proteinprepare­().^25^ A pure 1-palmitoyl-2-oleoylphosphatidylcholine (POPC) membrane 130 Å × 130 Å was built using the membrane builder class in HTMD. After that, each of the prepared proteins was embedded into a separate membrane. The systems are then solvated by adding a 20 Å layer of water molecules above and below the membrane and then neutralized to a 0.15 M NaCl concentration and built using the CHARMM builder in HTMD. The proteins, ions, and lipids were parameterized using the CHARMM36m^26^ force field, and TIP3P^27^ was used as the water model. The ligands were parameterized using the CGENFF.^28^ First, the correct mol2 file for each ligand was generated using the Schrodinger suite (Schrödinger Release 2023-3), and then the mol2 files were uploaded to the CGENFF web server to generate the parameters.

**Table 1:** Summary of Multiple Adaptively Sampled Trajectories^a^

systems		sampling time (μs)	number of trajectories
GLUT1	apo	12	300 ns × 40
	glucose	12	300 ns × 40
	urate	12	300 ns × 40
GLUT9	apo	78.6	300 ns × 262
	glucose	12	300 ns × 40
	urate	12	300 ns × 40
	apigenin	12	300 ns × 40

a Each trajectory is simulated at a time step of 0.1 ns.

A 40 ns NPT equilibration run at 310 K was performed for each system, with 5 kcal/mol/Å^2^ group restraints applied to the ligands with a box of 12 × 12 × 12 Å^3^ as a flat bottom to prevent the ligand from leaving the binding site during equilibration. The rest of the settings were kept the same as the default equilibration protocol in HTMD. In the production protocol, we ran the simulations in the *NVT* ensemble using a Langevin thermostat and a damping constant of 0.1 ps^–1^. The hydrogen mass repartitioning scheme was enabled to implement a 4 fs time step. During the production phase, all restraints were removed. For another set of simulations, the restraints box was expanded to allow the ligand to move inside the channel but not to diffuse into the solvent. An Adaptive Bandit-enhanced sampling protocol utilizing multiple short simulations based on Markov state models (MSMs) was employed. This adaptive algorithm iteratively conducts short parallel simulations, minimizing redundancy by discretizing the conformational space into an MSM and estimating free energy from the stationary distribution of each state. Simulations were restarted from low-energy conformations. The MetricSelfDistance function assessed native Cα contacts across residues to construct the MSMs, using an exploration value of 0.01 and a goal-scoring function of 0.3. Each round consisted of four 100 ns simulations, with trajectory frames recorded every 0.1 ns. The GLUT9 apo system was simulated for a cumulative 78.6 μs to achieve enough sampling to describe the opening and closure of either side of the transporter, while 12 μs was enough to separate conformations for other systems. All simulations were carried out using the ACEMD molecular dynamics engine.^24,29^

### Non-Markov State Model

The Integrative Generalized Master Equation (IGME)-based non-Markovian dynamic model was built based on a published IGME tutorial.^13^ Non-Markovian models like quasi-MSM overcome the data challenge of Markovian models, which require a long enough lag time to allow transition between states to become Markovian, by using the memory kernels.^30^ This allows much more precise predictions of slow dynamics from significantly short MD simulations.^30^ However, numerical instability becomes the major challenge of the qMSM. The recently introduced new IGME model solves this issue through the utilization of the integrals of memory kernels, thus reducing the numerical fluctuation.^31^ Two hyperparameters were used to build the IGME model: τ_k_ and *L*. τ_k_ is the memory kernel relaxation time, which should be long enough for the memory kernels to decay to zero. *L* is the length of the input data used in least-squares fitting. To fine-tune the hyperparameters, 100 IGME models were scanned for each system, and the best hyperparameters were selected based on the calculated root mean squared error (RMSE) between the IGME-predicted and MD-derived transition probability matrices (TPMs) across a range of lag times (Figure S12).

In this study, the χ_1_ angle of essential ligand-binding residues and the distance between helices were used as the input features of the IGME model (for details, see Supporting Information). Featurized trajectories were then projected onto three independent components (ICs) using time-lagged independent component analysis (tICA). An appropriate tICA lag time that gives a good separation of the projection was then selected for every system. K-means was then used to further cluster the data into 100 clusters based on the selected ICs in every system. The IGME model starts with a microstate MSM by using the clustered data. Then, PCCA + kinetic lumping was applied to convert the microstate MSM into a macrostate MSM with a selected number of states for every system. IGME then predicts a more accurate transition probability matrix (TPM) from the TPM of the macrostate MSM. The Chapman–Kolmogorov test was used to assess the quality of the IGME models. The mean first passage times (MFPT) between states were calculated with the row-normalized TPM. The net flux pathways between macrostates, which start from the highest energy state to the lowest energy state, were calculated using the transition pathway theory (TPT) function. For each metastable state, a structure that is closest to the k-means clustering center was selected and extracted as the representative conformation of this state. Moreover, all the frames belonging to each state were selected and merged using MDAnalysis for further analysis.^32,33^ Table 2 represents all of the parameters used in generating the IGME model.

**Table 2:** Parameters Used to Build the IGME Model in Each System^a^

system	tICA lag (ns)	components	clustering	MSM lag (ns)	*L* (ns)	τ_k_ (ns)
GLUT1-Apo	1	3	100	5	9.5	0.4
GLUT9-Apo	0.2	3	100	2	9.6	0.4
GLUT9-API	2	3	100	2	9.5	0.1

a *L* is the length of the input data used in least-squares fitting, and τ_k_ is the memory kernel relaxation time.

### PathDetect-SOM

During the MD simulation, glucose and urate exit the transporter via the intracellular side of both GLUT1 and GLUT9. Pathway analysis of these ligands was carried out using an artificial neuron network-based method called PathDetect-SOM.^14^ To train the model, residues that surround the intracellular side of the transporters were selected (details in Supporting Information). The time-dependent pairwise Euclidean distance between the selected residues and the ligand was automatically computed. This was then used as the input feature, which was used to train the self-organizing map (SOM) model iteratively. Each frame was considered as a data point and assigned to the most familiar neuron. These neurons were then grouped into an optimal number of clusters based on the silhouette profile. The representative structure of each neuron is saved using GROMACS,^34^ and for each cluster, the tool would identify a representative neuron. All the frames belonging to each neuron were extracted and merged using MDTraj.^35^ In this study, default 10 × 10 sheet-shaped SOMs without periodic boundary conditions were trained over 5000 training cycles. The distance capping was set to 12 Å, where distances exceeding the threshold would be ignored and considered as the capping value. The pathway identified by this tool can be directly illustrated by constructing the neuron transition network using an igraph. The heatmap, which contains the contact distance between the ligand and selected input residues across the frame of each cluster, was calculated via MDAnalysis.^32,33^

### Trajectory Analysis

Quantitative analysis of any conformational changes is based on the analysis of the whole frames extracted for each state and cluster. The χ_1_ angle distribution of the specific residue and the solvent accessible surface area (SASA) of each state are calculated using MDTraj.^35^ Distance distributions between either atoms or residues are computed using MDAnalysis.^32,33^ MDciao^36^ is utilized to investigate any changes in the residue–residue contact frequencies, residence times, and χ_1_ angle orientations.

## Supplementary Material



## Data Availability

The simulation data open access and is available at http://doi.org/10.5281/zenodo.14976303.

## Notes

Detailed information on constructed non-Markov state models and figures giving additional explanations to the main contents (PDF)
