# Supplementary material for: Structural Insights into the Substrate Egress Pathways Explains Specificity and Inhibition of Human Glucose Transporters (GLUT1 and GLUT9)
Source: ACS Pharmacol Transl Sci. 2025 May 15;8(6):1778–90. doi: 10.1021/acsptsci.5c00209 (PMC12171873; doi:10.1021/acsptsci.5c00209)
Supplement: Supplementary file 1 [file pt5c00209_si_001.pdf]

Supplementary Information

Structural Insights into the Substrate Egress Pathways Explains Specificity and Inhibition of Human Glucose Transporters (GLUT1 and GLUT9)

Manming Xu[1‡], Jiwen Jiang[1‡], Lin Gao[1], Saleh O Alyemni[1], and Shozeb Haider[1,2,3]*

[1] UCL School of Pharmacy, University College London, London WC1N 1AX, U.K

[2] University of Tabuk (PFSCBR), Tabuk, 71491, Saudi Arabia

[3] UCL Center for Advanced Research Computing, University College London, WC1H 9RL, U.K.

Prof Shozeb Haider (shozeb.haider@ucl.ac.uk)

ORCID: 0000-0003-2650-2925

Supplementary Information Text

Residues used in nMSM

In this study, essential residues involved in ligand binding within GLUT1 and GLUT9 were identified and selected according to previous studies[1, 2]. These critical residues were then superimposed from structural alignment to ensure consistent selection criteria across both proteins. In other words, if a key residue in GLUT1 did not have a corresponding essential residue in GLUT9, the aligned counterpart residue would be included to maintain uniformity in residue selection between the two structures. The residue selected in GLUT1 to calculate the X_1 angle are: T_{30}, S_{80}, L_{137}, R_{153}, Q_{161}, I_{164}, I_{168}, A_{171}, Q_{282}, Q_{283}, I_{287}, N_{288}, F_{291}, N_{317}, R_{333}, F_{379}, E_{380}, W_{388}, W_{412}. The residue selected in GLUT9 to calculate the X_1 angle are: L_{75}, T_{125}, L_{182}, R_{198}, A_{206}, F_{209}, V_{213}, G_{216}, Y_{327}, Q_{328}, L_{332}, N_{333}, W_{336}, E_{364}, R_{380}, F_{426}, C_{427}, F_{435}, W_{459}.

Except these residues participate in substrate transport, several residue pairs are also selected and their distance between Cαs is used to describe the helix movements in transporters. In GLUT1, residue pairs chosen are: G_{10} – I_{272}, V_{32} – A_{331}, P_{58} – P_{306}, F_{86} – R_{330}, R_{93} – R_{334}, L_{115} – M_{364}, F_{119} – V_{433}, V_{147} – L_{394}, R_{153} – P_{399}, G_{175} – C_{429}, L_{185} – L_{357} and F_{206} – F_{450}. While in GLUT9, the chosen residue pairs are: S_{55} – V_{313}, H_{97} – A_{346}, P_{103} – P_{350}, L_{135} – H_{377}, K_{138} – R_{380}, A_{161} – W_{410}, F_{164} – C_{480}, I_{192} – F_{441}, K_{195} – P_{448}, G_{220} – L_{476}, T_{230} – L_{404} and L_{249} – V_{498}.

Residues selected in PathDetect-SOM

GLUT1

T_{30}, F_{72}, S_{80}, T_{137}, V_{147}, T_{150}, R_{153}, Q_{161}, I_{168}, R_{212}, E_{247}, Q_{282}, Q_{283}, I_{287}, N_{288}, F_{291}, N_{317}, A_{331}, F_{379}, E_{380}, W_{388}, V_{391}, Q_{397}, P_{399}, I_{404}, W_{412}, and N_{415} were selected to calculate the Euclidean distances with substrates.

GLUT9

L_{75}, F_{117}, G_{125}, L_{182}, I_{192}, I_{195}, L_{198}, A_{206}, I_{209}, V_{213}, Y_{292}, T_{327}, Q_{328}, L_{332}, N_{333}, W_{336}, E_{364}, F_{426}, C_{427}, F_{435}, T_{438}, F_{444}, K_{451}, N_{458}, W_{459}, and N_{462} were selected to calculate the Euclidean distances with substrates.

Supplementary Figures

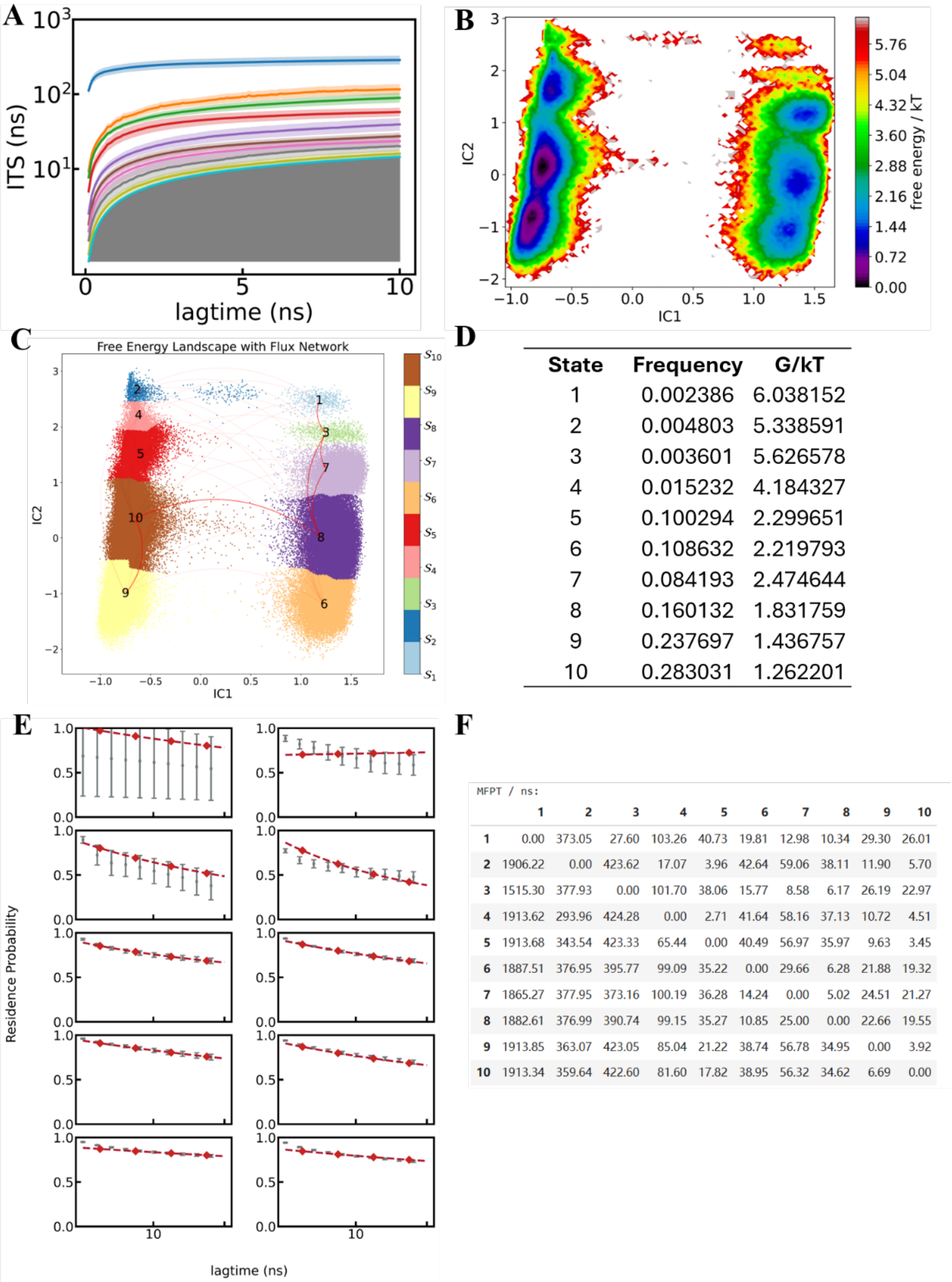

D

State	Frequency	G/kT
1	0.002386	6.038152
2	0.004803	5.338591
3	0.003601	5.626578
4	0.015232	4.184327
5	0.100294	2.299651
6	0.108632	2.219793
7	0.084193	2.474644
8	0.160132	1.831759
9	0.237697	1.436757
10	0.283031	1.262201

F

MFPT / ns:

	1	2	3	4	5	6	7	8	9	10
1	0.00	373.05	27.60	103.26	40.73	19.81	12.98	10.34	29.30	26.01
2	1906.22	0.00	423.62	17.07	3.96	42.64	59.06	38.11	11.90	5.70
3	1515.30	377.93	0.00	101.70	38.06	15.77	8.58	6.17	26.19	22.97
4	1913.62	293.96	424.28	0.00	2.71	41.64	58.16	37.13	10.72	4.51
5	1913.68	343.54	423.33	65.44	0.00	40.49	56.97	35.97	9.63	3.45
6	1887.51	376.95	395.77	99.09	35.22	0.00	29.66	6.28	21.88	19.32
7	1865.27	377.95	373.16	100.19	36.28	14.24	0.00	5.02	24.51	21.27
8	1882.61	376.99	390.74	99.15	35.27	10.85	25.00	0.00	22.66	19.55
9	1913.85	363.07	423.05	85.04	21.22	38.74	56.78	34.95	0.00	3.92
10	1913.34	359.64	422.60	81.60	17.82	38.95	56.32	34.62	6.69	0.00

Figure S1 – nMSM Model of GLUT1 Apo. **A.** Implied timescales (ITS) plot, a lag time of 5ns was chosen to build the nMSM. **B.** Free energy landscape projected by independent components. **C.** State distribution with flux network. **D.** Frequency and calculated free energy of each state. **E.** Chapman-Kolmogorov (CK) test plot. **F.** Mean first passage times (MFPT) between metastable states.

A

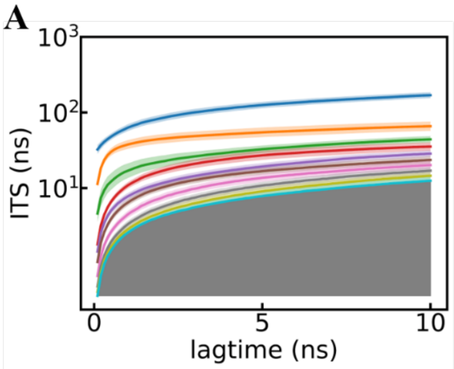

B

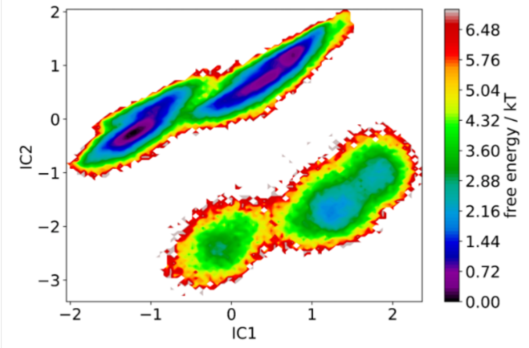

C

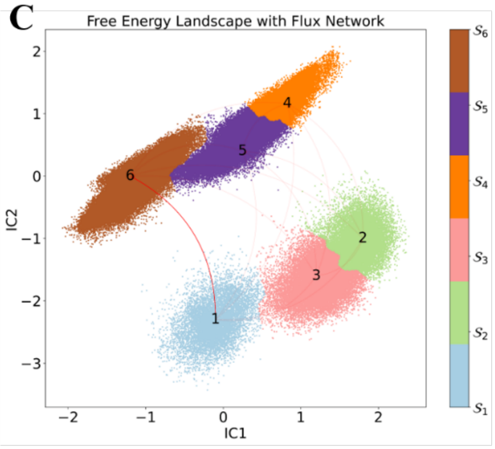

D

State	Frequency	G/kT
1	0.037004	3.296725
2	0.048813	3.019769
3	0.107617	2.229180
4	0.189058	1.665700
5	0.253163	1.373724
6	0.364346	1.009652

E

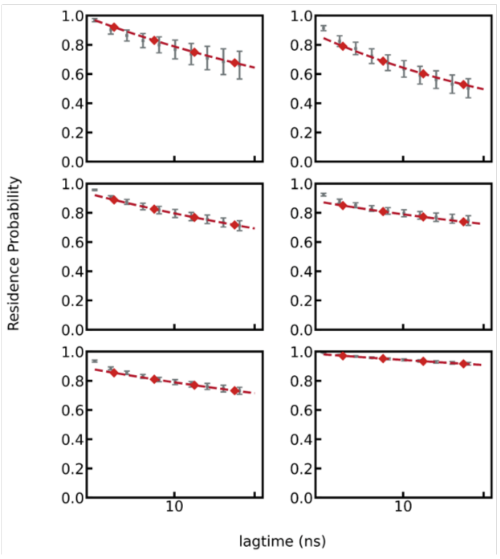

F

MFPT / ns:

	1	2	3	4	5	6
**1**	0.00	32.35	17.94	16.27	10.13	7.86
**2**	54.19	0.00	11.74	6.28	5.49	16.54
**3**	49.40	21.36	0.00	10.41	5.69	15.50
**4**	56.41	24.58	19.09	0.00	3.57	15.62
**5**	55.05	28.58	19.16	8.35	0.00	13.41
**6**	49.24	36.09	25.43	16.86	9.87	0.00

Figure S2 – nMSM Model of GLUT9 Apo. A. Implied timescales (ITS) plot, a lag time of 5ns was chosen to build the nMSM. **B.** Free energy landscape projected by independent components. **C.** State distribution with flux network. **D.** Frequency and calculated free energy of each state. **E.** Chapman-Kolmogorov (CK) test plot. **F.** Mean first passage times (MFPT) between metastable states.

A

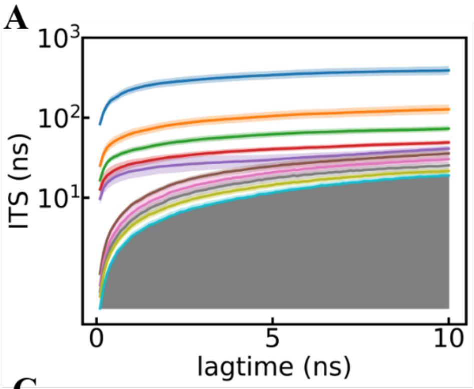

B

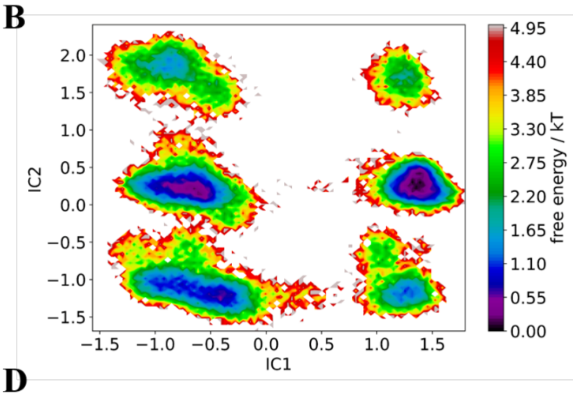

C

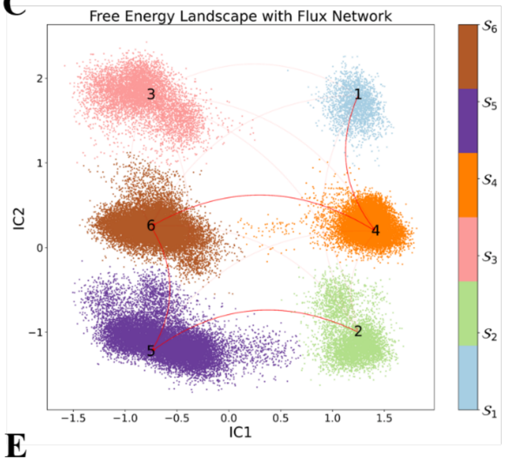

D

State	Frequency	G/kT
1	0.036633	3.306797
2	0.086933	2.442614
3	0.108733	2.218857
4	0.225167	1.490914
5	0.263533	1.333575
6	0.279000	1.276543

E

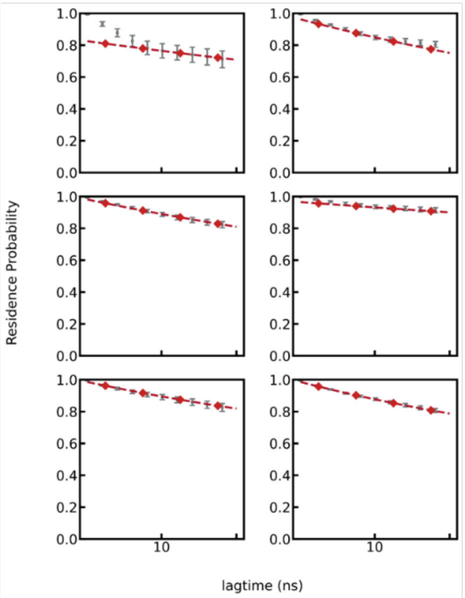

F

MFPT / ns:

	1	2	3	4	5	6
**1**	0.00	6.08	13.59	1.15	6.47	6.76
**2**	17.00	0.00	11.73	7.41	3.52	4.86
**3**	23.14	10.37	0.00	13.16	3.22	1.14
**4**	10.88	6.22	13.33	0.00	6.30	6.49
**5**	21.97	8.11	9.16	12.08	0.00	2.28
**6**	22.38	9.57	7.21	12.38	2.40	0.00

Figure S3 – nMSM Model of GLUT9 API. A. Implied timescales (ITS) plot, a lag time of 5ns was chosen to build the nMSM. **B.** Free energy landscape projected by independent components. **C.** State distribution with flux network. **D.** Frequency and calculated free energy of each state. **E.** Chapman-Kolmogorov (CK) test plot. **F.** Mean first passage times (MFPT) between metastable states.

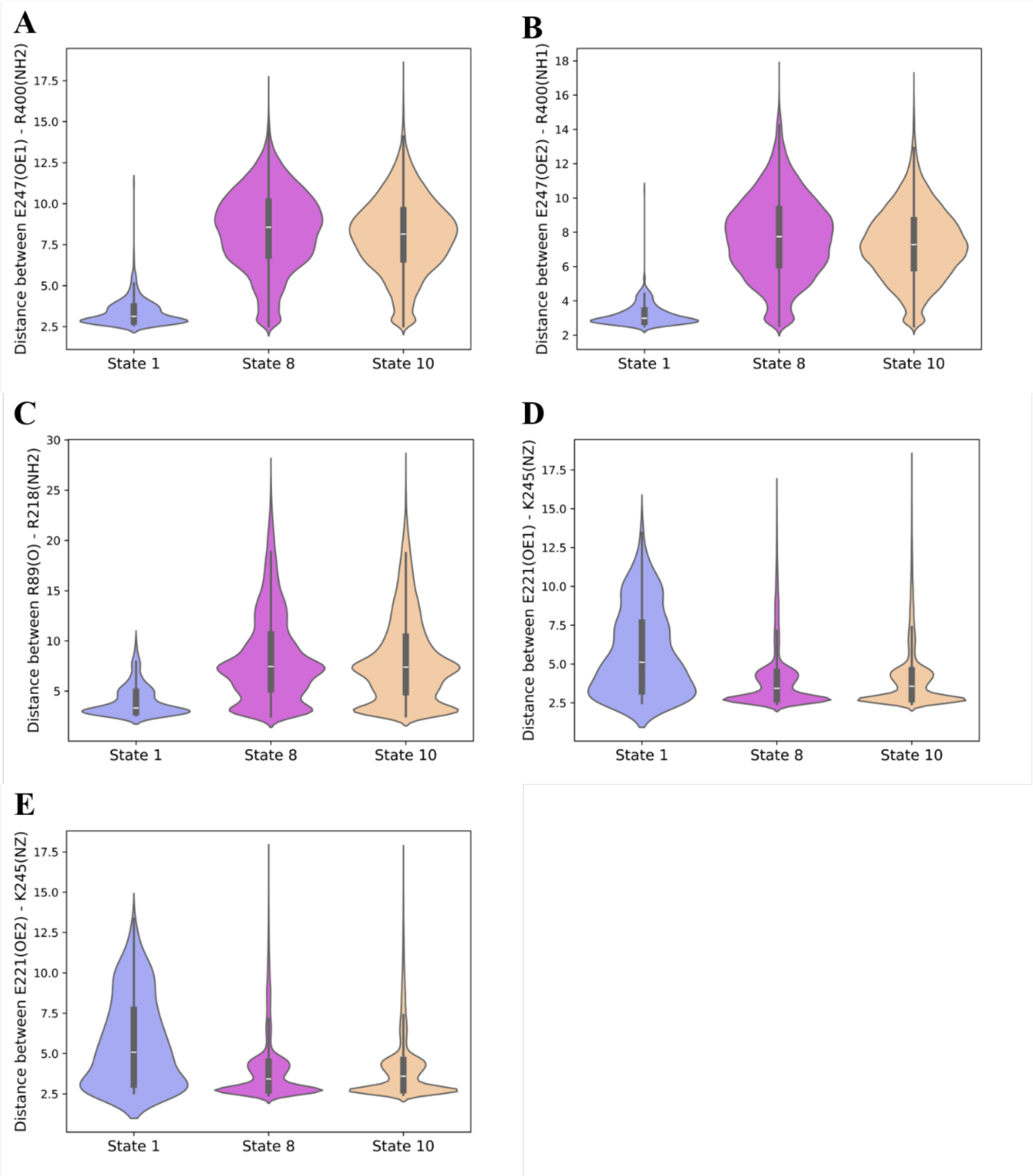

Figure S4 – Violin Plot of Distance Distribution Between Atoms of State 1, State 8, and State 10 (GLUT1 Apo). A. Distance between E_{247}(OE1) and R_{400}(NH2). **B.** Distance between E_{247}(OE2) and R_{400}(NH1). **C.** Distance between R_{89}(O) and R_{218}(NH2). **D.** Distance between E_{221}(OE1) and K_{245}(NZ). **E.** Distance between E_{221}(OE2) and K_{245}(NZ).

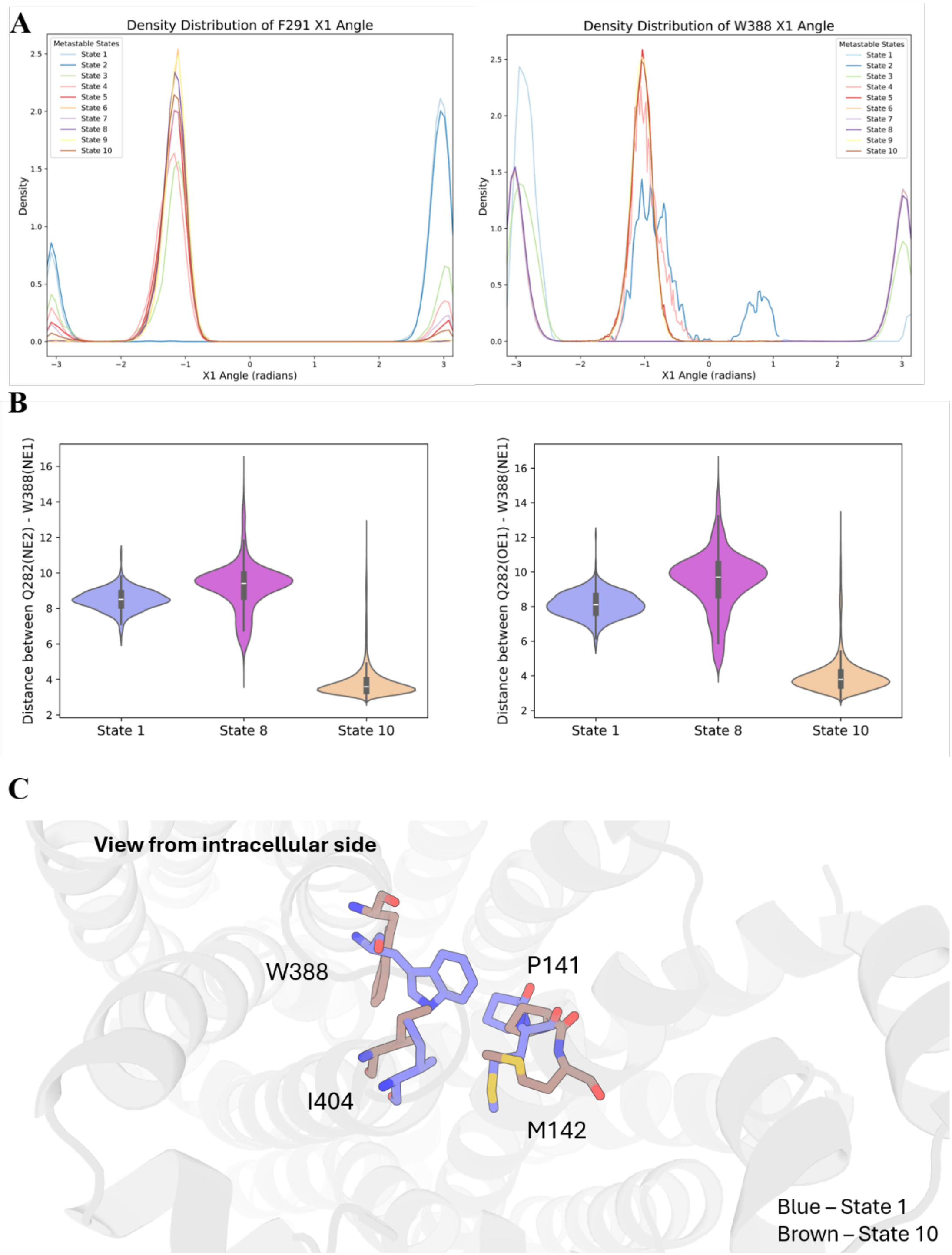

Figure S5 – Additional Figures for GLUT1 Apo. A. The distribution of the X_1 angle of F_{291}. **B.** The distribution of the X_1 angle of W_{388}. **C.** Distance distribution of Q_{282}(NE2) and Q_{282}(OE2) to W_{388}(NE1). State 10 shows strong interactions between these two residues. **D.** The hydrophobic gate formed by W_{388}, P_{141}, M_{142}, and I_{404}. Blue represents the conformation in state 1 and brown represents the conformation in state 10. State 10 is showing a wider tunnel with the sidechain of W_{388} being lifted towards the extracellular side.

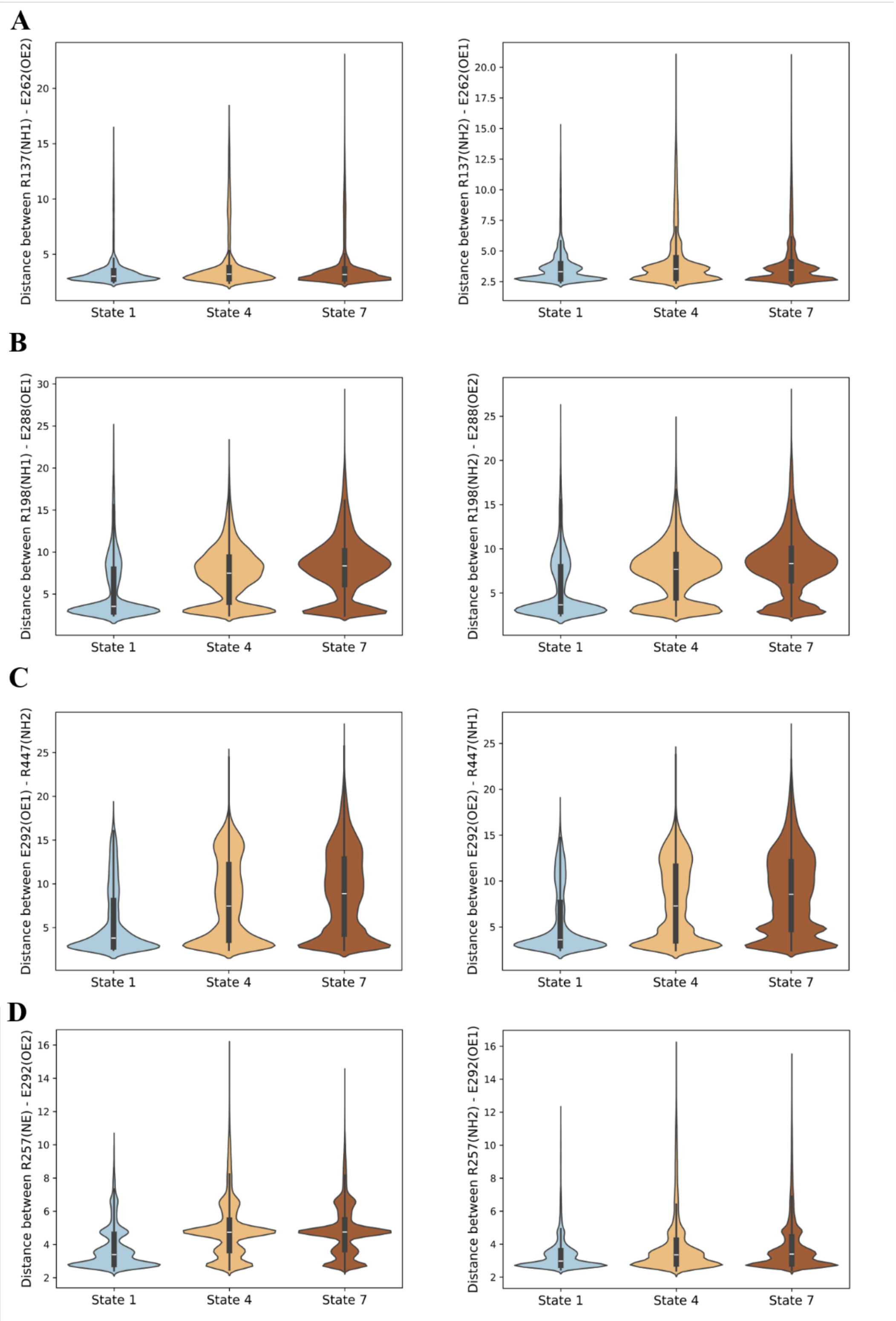

Figure S6 – Distance Distribution Between Residues in Each State (GLUT9 Apo).
A. R_{137} and E_{262}. **B.** R_{198} and E_{288}. **C.** E_{292} and R_{447}. **D.** R_{257} and E_{292}.

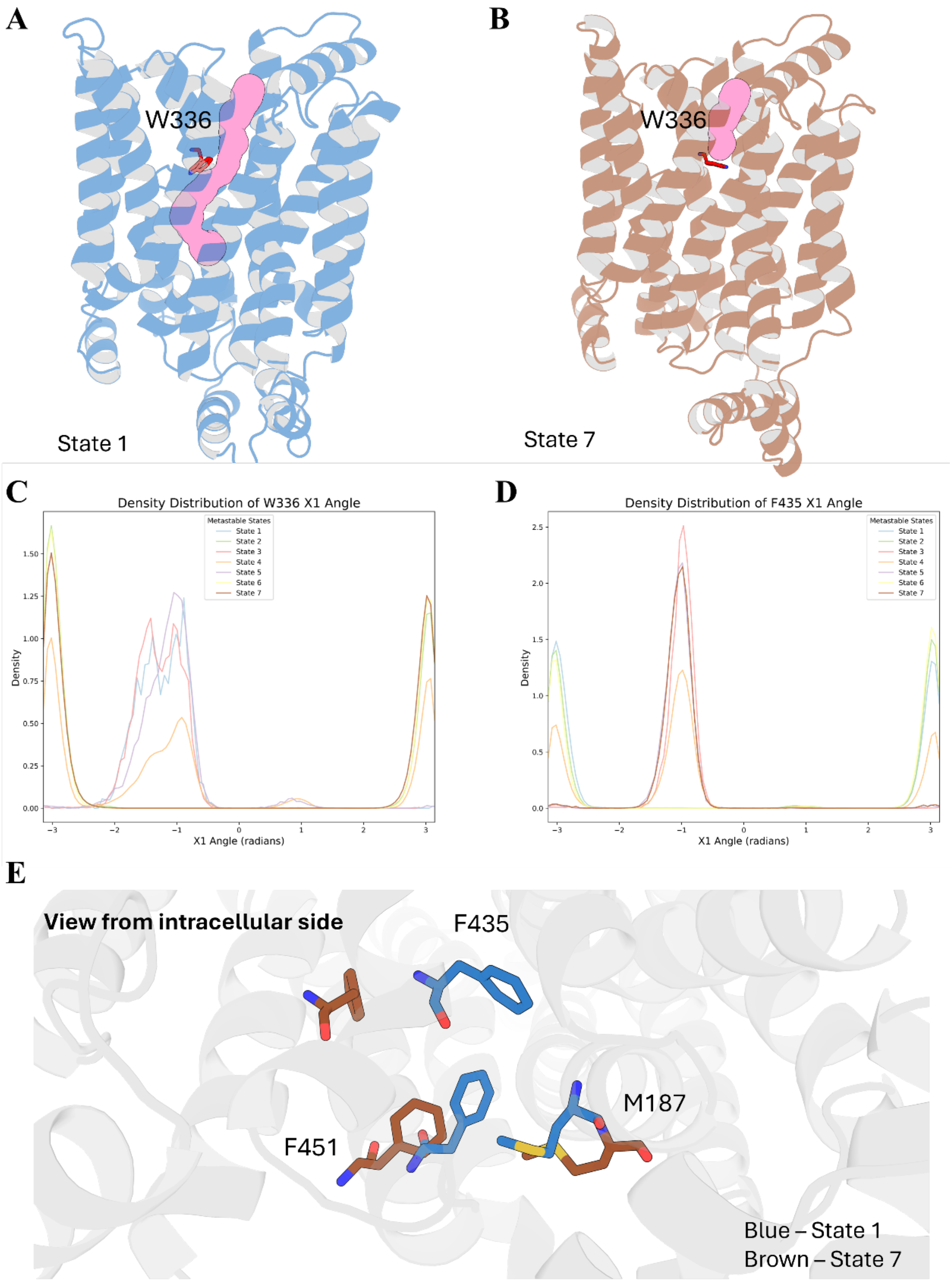

Figure S7 – Additional Figures for GLUT9 Apo. A&B. Tunnel Detected by CAVER[3] of State 1 (Blue) and State 7 (Brown). **C.** The distribution of the X_1 angle of W336. **D.** The distribution of the X_1 angle of F435. **E.** The hydrophobic core formed by M187, F451, and F435. Blue represents the conformation in state 1 and brown represents the conformation in state 7. State 7 shows a much wider tunnel with the orientation and movement of F435.

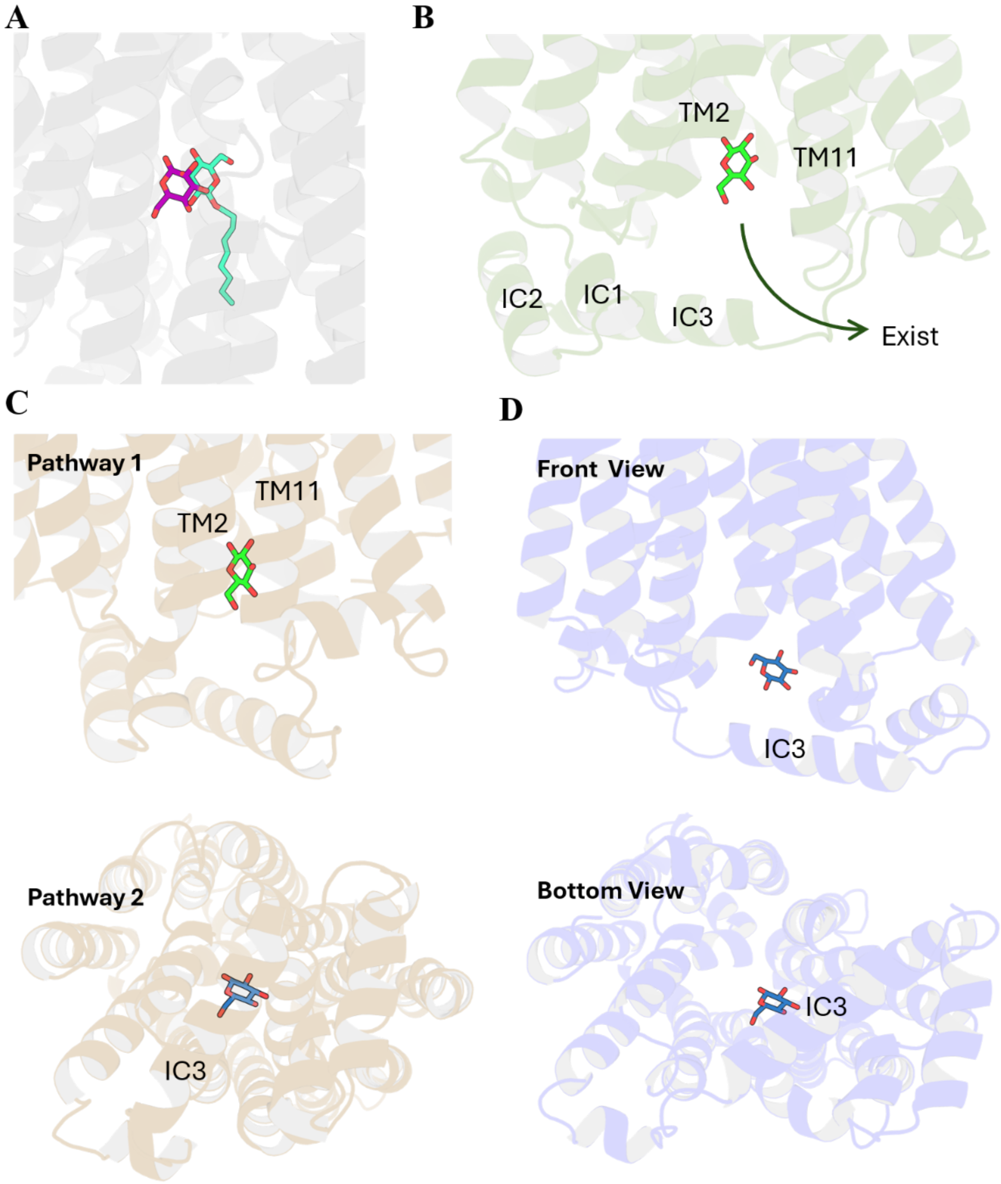

Figure S8 – Additional Figures for Glucose Pathway in GLUT1. A. Glucose binding conformation comparison between the binding state (Cluster E) to the X-ray crystallography binding structure (PDB id: 4PYP). **B.** Detailed position of glucose before existence in pathway 1 (Cluster B). **C.** Superimposition of glucose to the inward close conformation determined by nMSM (state 1) to determine whether the pathway still exists when the intracellular side is closed. **D.** Front view and bottom view of the position of glucose before existence in pathway 2 (Cluster G).

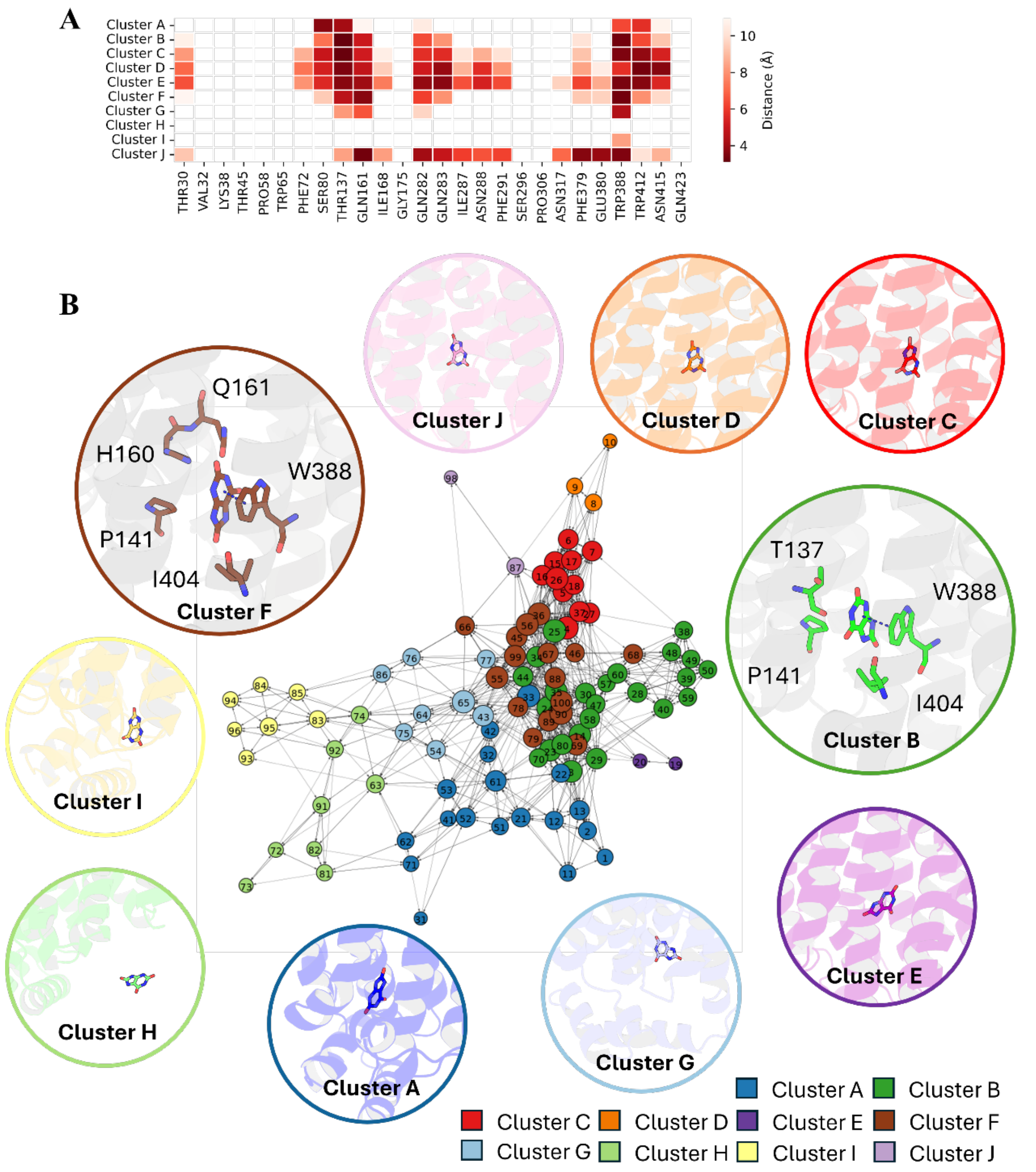

Figure S9 – GLUT1 Urate Pathway. A. Distance heatmap between urate and selected input residues during SOM training. **B.** Neuron transition network. Detailed ligand-protein contacts in each cluster are placed close to the neuron.

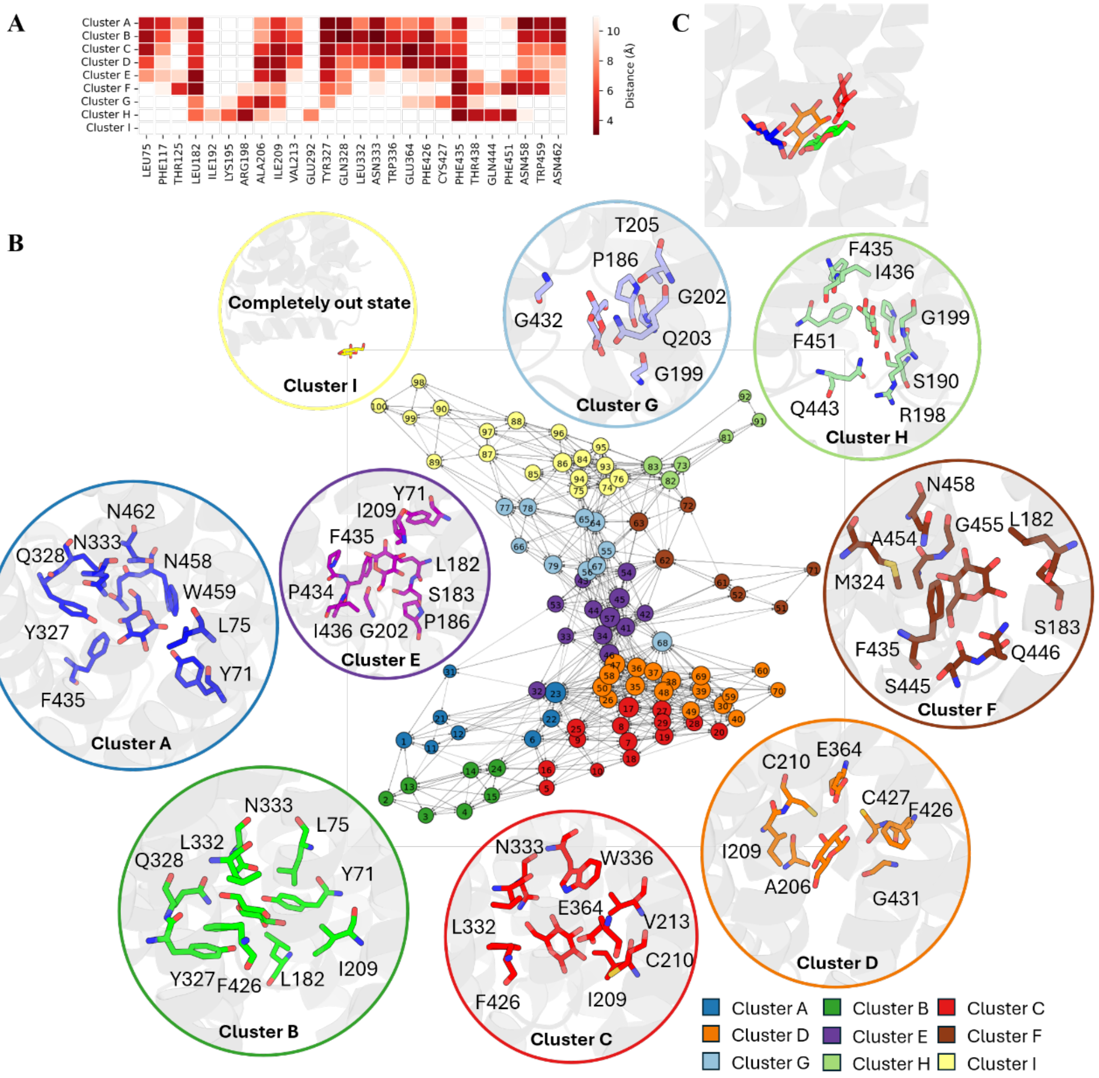

Figure S10 – GLUT9 Glucose Pathway. A. Distance heatmap between glucose and selected input residues during SOM training. **B.** Neuron transition network. Detailed ligand-protein contacts in each cluster are placed close to the neuron. **C.** Superimposition of glucose position in clusters A, B, C, and D.

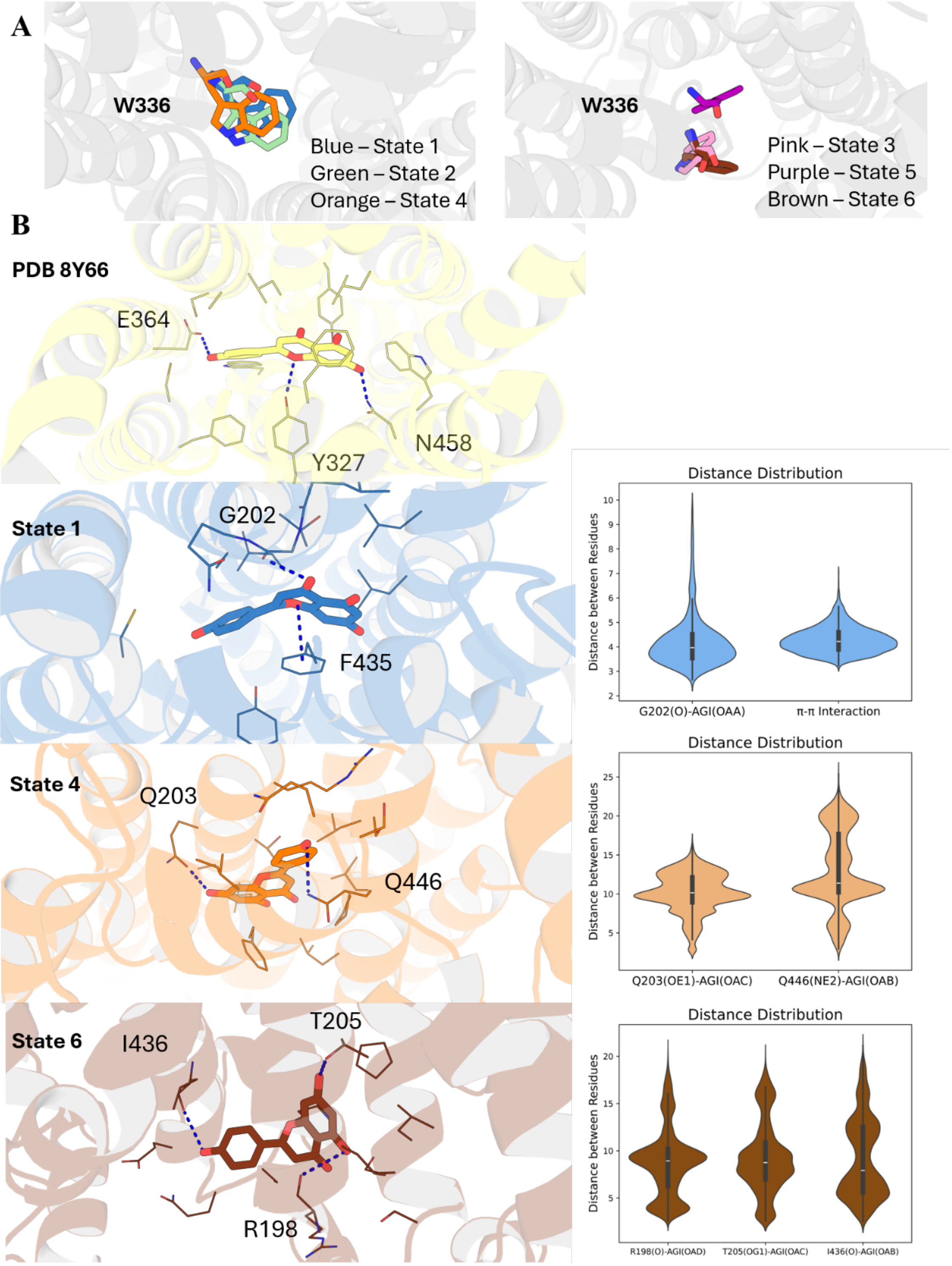

Figure S11 – GLUT9 API nMSM Additional Figures. A. Conformation of W_{336} in each state. **B.** Ligand binding position of Apigenin in the extracted representative structure of state 1, state 4, and state 6 with the calculated distance distribution among all the frames of each state.

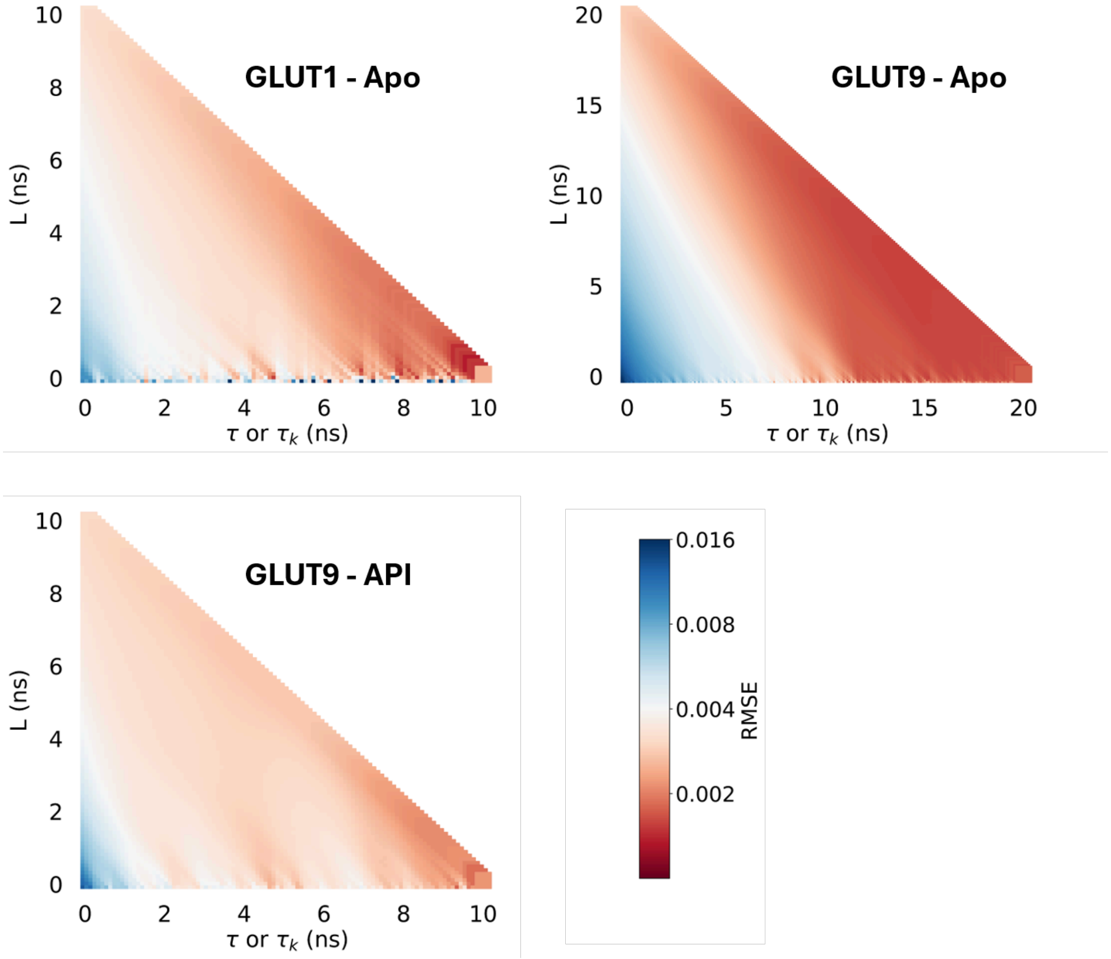

Figure S12 – Calculated root mean squared error (RMSE) of each IGME model.

Reference

(1) Deng, D.; Xu, C.; Sun, P.; Wu, J.; Yan, C.; Hu, M.; Yan, N. Crystal structure of the human glucose transporter GLUT1. *Nature* **2014**, *510* (7503), 121-125. DOI: 10.1038/nature13306

(2) Shen, Z.; Xu, L.; Wu, T.; Wang, H.; Wang, Q.; Ge, X.; Kong, F.; Huang, G.; Pan, X. Structural basis for urate recognition and apigenin inhibition of human GLUT9. *Nat Commun* **2024**, *15* (1), 5039. DOI: 10.1038/s41467-024-49420-9

(3) Chovancova, E.; Pavelka, A.; Benes, P.; Strnad, O.; Brezovsky, J.; Kozlikova, B.; Gora, A.; Sustr, V.; Klvana, M.; Medek, P.; et al. CAVER 3.0: a tool for the analysis of transport pathways in dynamic protein structures. *PLoS Comput Biol* **2012**, *8* (10), e1002708. DOI: 10.1371/journal.pcbi.1002708
